# Ediacaran *Corumbella* has a cataphract calcareous skeleton with controlled biomineralization

**DOI:** 10.1016/j.isci.2022.105676

**Published:** 2022-11-25

**Authors:** Gabriel Ladeira Osés, Rachel Wood, Guilherme Raffaeli Romero, Gustavo Marcondes Evangelista Martins Prado, Pidassa Bidola, Julia Herzen, Franz Pfeiffer, Sérgio Nascimento Stampar, Mírian Liza Alves Forancelli Pacheco

**Affiliations:** 1 Programa de Pós-Graduação em Ecologia e Recursos Naturais, Universidade Federal de São Carlos, Rodovia Washington Luís, Km 235, São Carlos-SP 13565-905, Brazil; 2 School of GeoSciences, University of Edinburgh, James Hutton Road, Edinburgh EH9 3FE, UK; 3 Laboratório de Paleobiologia e Astrobiologia, Universidade Federal de São Carlos, Rodovia João Leme dos Santos, Km 110, Sorocaba-SP 18052-780, Brazil; 4 Programa de Pós-Doutorado, Instituto de Física, Universidade de São Paulo, Rua do Matão, 1371, São Paulo-SP 05508-090, Brazil; 5 Instituto de Geociências, Universidade de São Paulo, Rua do Lago, 562, São Paulo-SP 05508-080, Brazil; 6 Programa de Pós-Graduação em Geoquímica e Geotectônica, Instituto de Geociências, Universidade de São Paulo, Rua do Lago, 562, São Paulo-SP 05508-080, Brazil; 7 Institute of Materials Physics, Helmholtz-Zentrum Hereon, Max Plank Straße 1, 21502 Geesthacht, Germany; 8 Research Group of Physics of Biomedical Imaging, School of Natural Sciences, Technical University of Munich, James-Franck Straße 1, 85748 Garching b. München, Germany; 9 Munich Institute of Biomedical Engineering, Technical University of Munich, Boltzmannstr. 11, 85748 Garching b. München, Germany; 10 Chair of Biomedical Physics, Department of Physics, School of Natural Sciences, Technical University of Munich, James-Franck Straße 1, 85748 Garching b. München, Germany; 11 Department of Diagnostic and Interventional Radiology, School of Medicine and Klinikum rechts der Isar, Technical University of Munich, Ismaninger Straße 22D, 81675 Munich, Germany; 12 Laboratório de Evolução e Diversidade Aquática, Departamento de Ciências Biológicas, Faculdade de Ciências - Câmpus de Bauru, Universidade Estadual Paulista, Av. Eng. Luiz Edmundo Carrijo Coube, 14-01, Bauru-SP 17033-360, Brazil

**Keywords:** Biological sciences, evolutionary biology, evolutionary processes

## Abstract

*Corumbella* is a terminal Ediacaran tubular, benthic fossil of debated morphology, composition, and biological affinity. Here, we show that *Corumbella* had a biomineralized skeleton, with a bilayered construction of imbricated calcareous plates and rings (sclerites) yielding a cataphract organization, that enhanced flexibility. Each sclerite likely possessed a laminar microfabric with consistent crystallographic orientation, within an organic matrix. Original aragonitic mineralogy is supported by relict aragonite and elevated Sr (mean = ca. 11,800 ppm in central parts of sclerites). In sum, the presence of a polarisation axis, sclerites with a laminar microfabric, and a cataphract skeletal organization reminiscent of early Cambrian taxa, are all consistent with, but not necessarily indicative of, a bilaterian affinity. A cataphract skeleton with an inferred complex microstructure confirms the presence of controlled biomineralization in metazoans by the terminal Ediacaran, and offers insights into the evolution of development and ecology at the root of the ‘Cambrian radiation’.

## Highlights

• *Corumbella* had a calcareous skeleton, with a cataphract organization of sclerites
• An original aragonitic mineralogy is supported by relict aragonite and elevated Sr
• Morphology and crystallographic orientation indicate controlled biomineralization
• Morphology and laminar microfabric are reminiscent of early Cambrian taxa

## Graphical abstract

**Figure:**
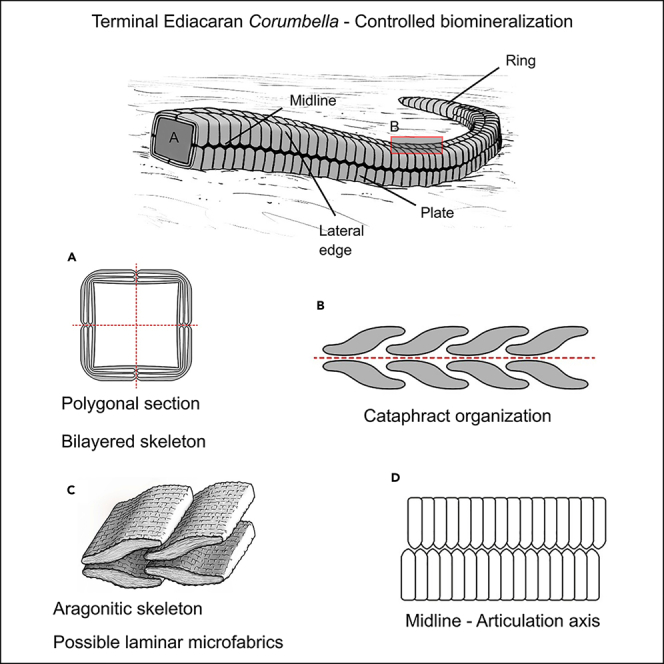

## Introduction

Metazoans developed skeletal, calcareous, hard parts in the terminal Ediacaran ca. 550 million years ago, probably independently in unrelated clades,^1^^,^^2^ representing a visible root of the ‘Cambrian radiation’. Much uncertainty remains, however, as to the affinity of the early biomineralizing taxa, the degree of biological control exerted over biomineralization, and their relationship to lower Cambrian taxa.

*Corumbella* is an extinct genus of terminal Ediacaran age represented by a single species, *Corumbella werneri,* known from siliciclastic and carbonate settings from Brazil, Paraguay, and the USA.^3^^,^^4^^,^^5^^,^^6^ *Corumbella* has been described as a flexible, tubular form with oral-aboral axis and 4-fold symmetry constructed by rings, and midlines formed by the junction of alternate continuous rings^5^^,^^6^^,^^7^^,^^8^ (Table S1; Figure S1). Rings have been assumed to have been formed by lamellae of either organic or weakly mineralized composition.^6^ *Corumbella* has been interpreted as epibenthic, with a point of substrate attachment and shows directional growth.^5^^,^^6^ The taxon has been attributed to either scyphozoan cnidarians related to the extinct conulariids^6^^,^^7^^,^^8^^,^^9^, or sinotubulitid or siboglinid annelids^10^ (Tables S2, S3, and S4).

Much uncertainty as to the morphology and biomineralization status of *Corumbella* is due to poor preservation of key features resulting from strong diagenetic overprinting. It has been suggested that *Corumbella* was biomineralized, but this is based only on the calcitic preservation of the rings.^6^^,^^10^^,^^11^ It has not been established whether this calcite is a diagenetic replacement of an organic precursor skeleton, or of a different original, but unstable, carbonate polymorph.

Here we describe exceptionally preserved material from fine-grained marls and shales of the late Ediacaran Tamengo Formation, ca. 555-542 Ma^12^ from Corumbá and Ladário, Brazil (Figure S2; Data S1). We apply light petrography coupled with cathodoluminescence (CL) imaging, micro-Raman spectroscopy, scanning electron microscopy (SEM), including backscattered electron imaging (BSE), energy-dispersive X-ray spectroscopy (EDS), electron backscatter diffraction (EBSD), and electron microprobe analysis (EPMA), and X-ray microtomographic (micro-CT) imaging, to resolve the morphology and composition of *Corumbella.* This multi-technique approach enables the formulation of a morphological reconstruction to be presented, reminiscent of early Cambrian taxa with cataphract skeletal organization, defined as articulating imbricated elements. Our data further demonstrate that biomineralization in *Corumbella* was biologically-controlled, therefore informing the evolution of controlled biomineralization and its ecological implications.

## Results

### Optical microscopy

**Figure 1:**
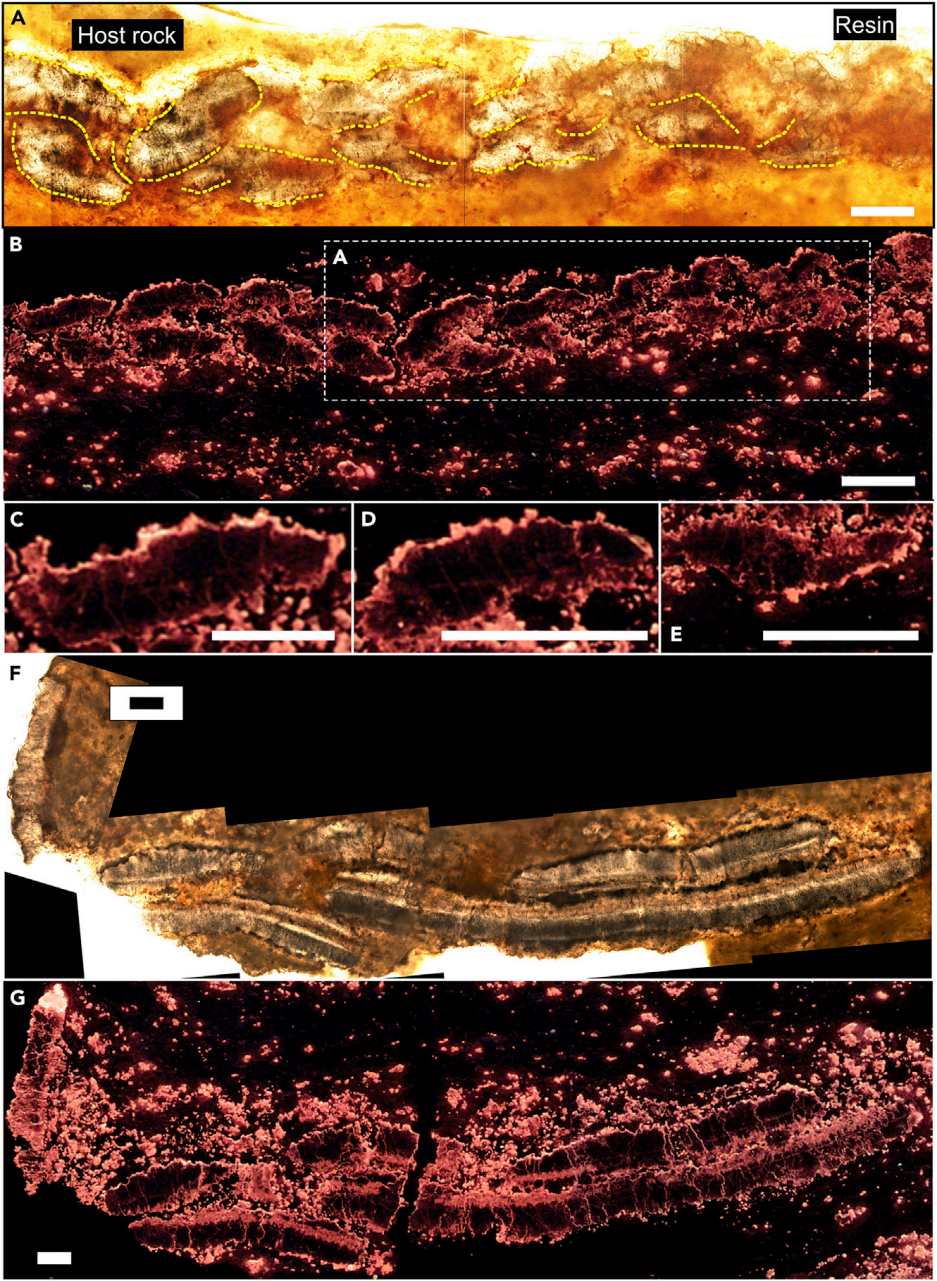
Photomicrographs of highly polished thin sections showing the skeletal structure of *Corumbella werneri* (A–E) Sample CAP/1F 11. F and G: Sample CAP/1F 12. The cutting direction of original samples is available in Figures S3A and S3C. (A) Plane polarised light (PPL) image of tube longitudinal section, showing calcitic plates outlined by Fe-(oxyhydr)oxides film (dashed yellow lines). (B) Cathodoluminescence (CL) image of two layers of imbricated plates. Area shown in A highlighted. (C–E) CL images of individual plates. F and G: Respectively, PPL and CL images of the cross-section of tube showing plates. Scale bars: A, C, F, and G = 0.1 mm; B, D, and E = 0.2 mm. The assembling of original figures resulting in A-G is shown in Figure S6. Original figures have been cropped to remove excess of empty space around the fossils and also to enable stitching.

Light photomicrographs of longitudinal sections of the *Corumbella* tube show crystalline areas of calcite surrounded by red staining (Figure 1A; see Figure S3A for original sample).

### Cathodoluminescence imaging

**Figure 2:**
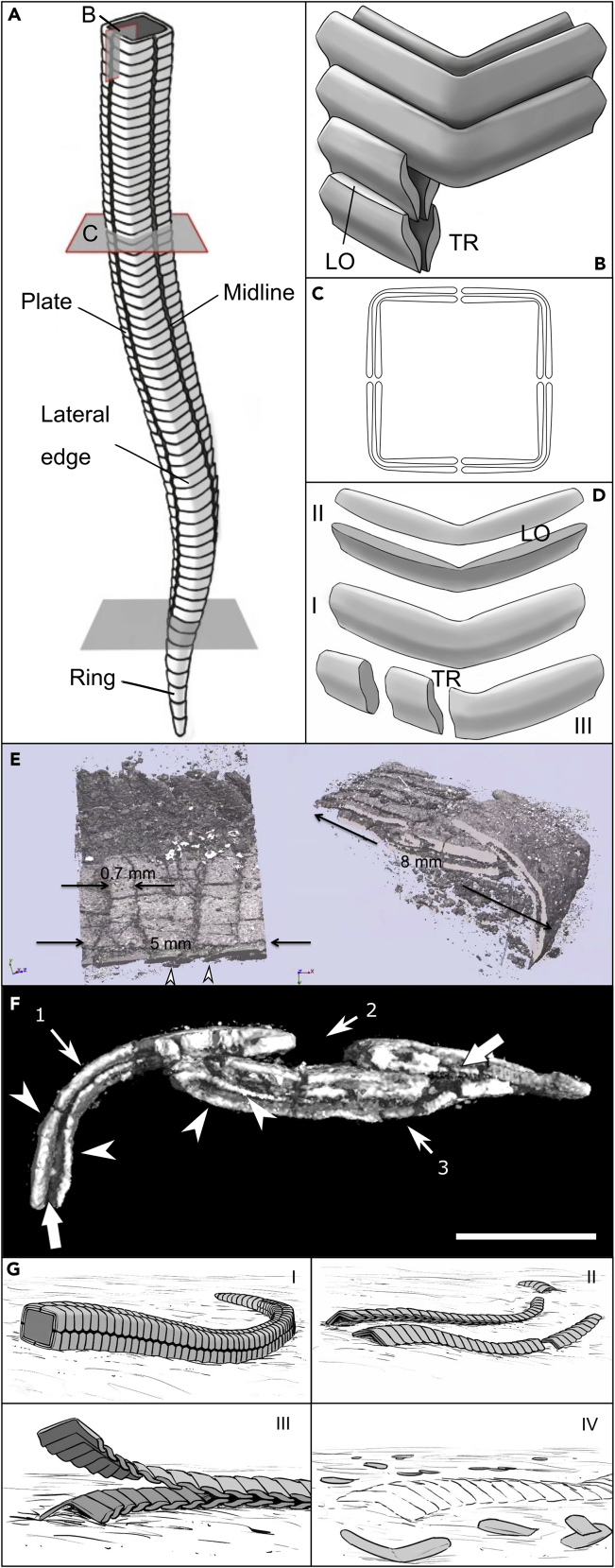
Morphological reconstruction, micro-CT, and disarticulation patterns of *Corumbella* skeleton microstructure Sample GP/1E 574a in E and F. (A) Hypothesis for the reconstruction of an individual. The lowermost gray plane marks the transition from the proximal to the distal parts, which are respectively build by rings and plates. (B) Cataphract organization of regularly imbricated plates in two layers. “LO” and “TR”, respectively longitudinal and transverse sections of sclerites. (C) Tube cross-section with the longitudinal section of plates. (D) Plate (I), respectively in longitudinal (II) and in transverse section (III). “LO” and “TR”, respectively longitudinal section and transverse section. (E) 3D rendering of a 3D specimen in slightly rotated plane view (left), with plate imbrication shown at the image bottom (arrowheads); and in the cross-section of the tube (right). Modified from Figure 6B of Pacheco et al.^6^ (https://doi.org/10.1371/journal.pone.0114219), originally published under a Creative Commons Attribution License. (F) 3D rendering (showing cross-section of the tube) of the same specimen in E. The direction of sectioning is shown in Figure S7. Arrows indicate the gap between two stacked skeletal layers; arrowheads indicate the bilayered skeletal wall; 1-Lateral edge, 2-Midline in cross-section, and 3-Collapsed wall. Scale bar: 2 mm. F was modified from Figure 3D of Bidola et al.^89^; permission for electronic reuse granted by “Cambridge University Press” via RightsLink. The full 3D rendering of sample GP/1E 574a is available in Video S1. (G) Types of disarticulation shown in Figure S8. I-Articulated specimen; II-Disarticulation along midline and between plates and rings; III-‘Peeling’, that is disarticulation between layers of sclerites; and IV-Disarticulation of consecutive sclerites (plates) leaving molds and plates fragmented at the lateral edge. A and F original figures have been cropped to remove excess of empty space.

Cathodoluminescence (CL) and 3D micro-CT imaging, which has been used to investigate the 3D morphology of the skeleton, reveals the tubes of these organisms to be comprised of two layers of discontinuous plates and rings, together termed sclerites, that are imbricated in opposite directions in each layer (Figures 1B–1E, 2A–2F, S4, and S5). Each sclerite has rounded or pointed terminations and a sigmoidal shape (Figures 1B–1E, 2B, 2D, and 2E). The inner tube wall is not smooth due to the imbrication of the inner layer (Figure 1B). Plates and rings appear to have irregular surfaces (Figures 1B–1E and S4). A single thicker element lies close to the proximal region of the tube (Figures S4A and S4B). In plane view, each plate progressively tapers toward its terminations at the main axis of tube faces, along which plates are displaced on either side of the midline articulation axis^5^ (Figures 2A and S3A). Cathodoluminescence was used to further investigate the composition of the skeleton. Under CL, the skeleton shows a patchy but generally non-to-dull luminescence but with brightly luminescent margins (Figures 1B–1E, 1G, and S4B). The innermost margins of the skeleton may also show a more continuous brightly luminescent layer (Figure 1G). This layer may also be present within the central part of the skeleton, and some parts appear to be composed of vertically elongated, palisade-like sparry crystals at their margins (Figure S4F).

In the transverse section of the polyhedral tube, the plates become narrow at the continuous lateral edge (Figures 2C, 2E, and 2F; Video S1). In plane view, the midlines are convergence zones of two alternating individual plates (Figure S3A). We, therefore, interpret the gap close to the center of a face as the midline cross-section (Figure 2F). Some specimens apparently show more than two layers, but this is duplication due to fragmentation and folding after burial compression (Figures 1F, 1G, and 2F; Video S1).

Video S1. 3D rendering of micro-CT with synchrotron light source of Sample GP/1E 574a, related to Figure S7 Scale in Figure 2E.

### Raman spectroscopic, scanning electron microscopy, and energy-dispersive X-Ray spectroscopy

**Figure 3:**
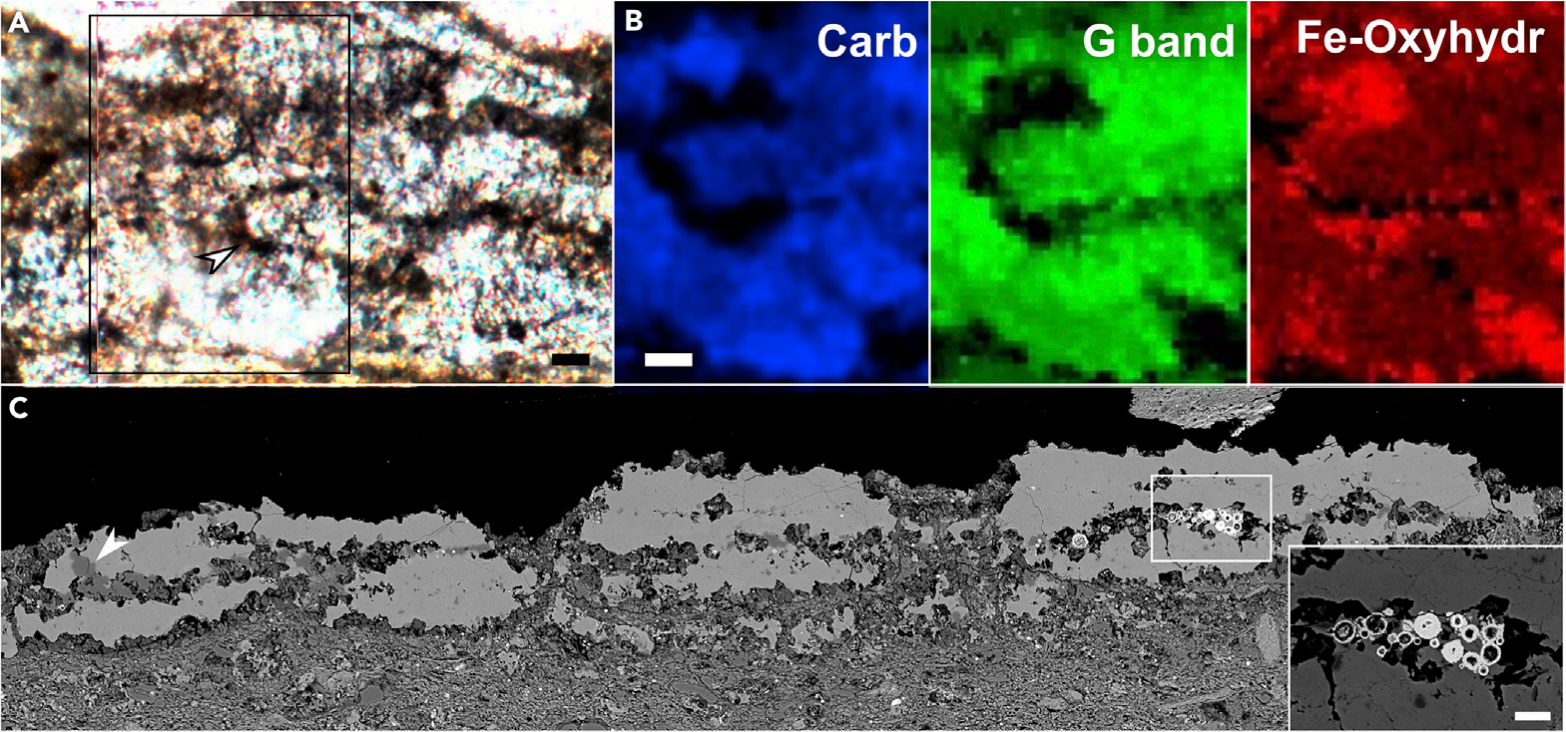
Micrographs of microstructure of *Corumbella* skeleton, Raman mapping and scanning electron microscopy (SEM) images Samples CAP/1F 14 (A) and CAP/1A 1022 (C). The cutting direction of the original samples that yielded sections in A and C is available in Figures S3B and S3D. The area shown in C is delimited in Figure S4B. (A) PPL image of tube longitudinal section, with inset showing region imaged with Raman maps (B) - carb-carbonate, G band of amorphous carbon and Fe-(oxyhydr)oxides. Arrowhead indicates Fe-(oxyhydr)oxides film in A. (C) Backscattered electron (BSE) image of tube longitudinal section showing partially silicified rings (arrowhead-dark gray areas) and pyrite pseudoframboids (inset) between ring layers. Scale bars: 20 μm. (A-C) original figures have been cropped to remove excess empty space.

Raman spectroscopic mapping was employed to determine the composition of the skeleton. This analysis reveals that both calcite and amorphous carbon are abundant throughout the sclerites (Figures 3A and 3B; Table S5; see Figure S3 for original sample). The red staining surrounding the skeletal margin is revealed to be composed by Fe-(oxyhydr)oxide (Figures 1A, 3A, and 3B), particularly oxidized pyrite framboids, as shown by SEM imaging and EDS spectra, and localized areas of the skeleton are silicified (Figures 3C and S9).

### Electron backscatter diffraction

**Figure 4:**
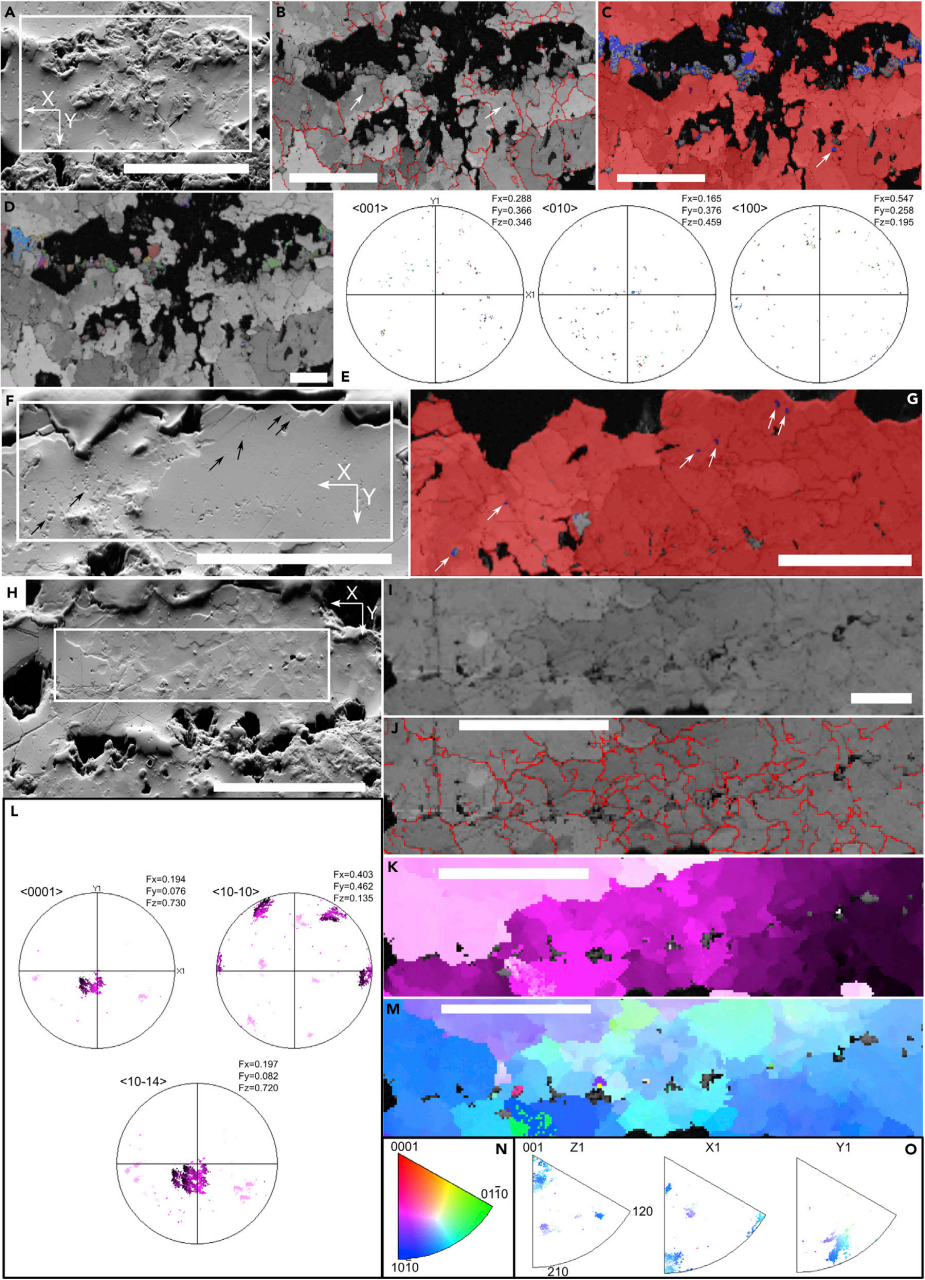
Original mineralogy and mineralogical texture of *Corumbella* sclerites (rings) shown by electron backscatter diffraction (EBSD) All measurements in the tube longitudinal section in Sample CAP/1A 1022 (Figures S3B and S4). (A, F, and H) SEM images. Reference directions X and Y are shown. The delimited areas (Figure S4) correspond, respectively, to B-D, G, and I-K/M. B, I, and J: Band contrast images (grain boundaries as red lines). Arrows point to intracrystalline dissolution in B. (C and G) Phase contrast maps of calcite (red) and aragonite (blue). Arrows indicate aragonite relicts shown in the correspondent images A and F (arrows). (D and K) Crystallographic orientation maps, respectively, for aragonite and calcite. (E and L) Pole figures of the orientation, respectively, of aragonite and calcite crystals correspondent to maps D and K, respectively. (M) Inverse pole figure (IPF) orientation map. (N) Color code of map in M. (O) IPFs with color code in M. Scale bars: A, F, and H = 100 μm; B, C, G, J, K, and M = 50 μm; D and I = 20 μm. B, D-F, J-L, and O original figures have been cropped to remove excess empty space.

EBSD was used to further investigate the morphology, composition, and crystallography of crystals forming the skeleton. This analysis shows that the skeleton is formed by subhedral prismatic, anhedral microspar, and sparry calcite crystals with mosaic, and subordinately, palisade textures at the sclerite margin (Figures 4B, 4C, 4G, 4I, 4J, S4B, and S4F). Diagenetic dissolution of the terminations of such crystals creates the apparently irregular surface of the sclerites, and alteration yields the brightly luminescent margins (Figure S4B). Intracrystalline dissolution is also noted within crystals (Figure 4B).

EBSD analysis shows that sparry calcite crystals have a dominant c-axis orientation, with a limited variability, at a low angle to the long axis of *Corumbella*, almost parallel to the sclerite surface at the measurement point (Figure 4L). The pole figure of <10-10 > axes has three pole maxima and the pole figure of <10-14 > axes has a repetition of each domain, both patterns associated with calcite symmetry (Figure 4L). The distribution of axes and corresponding crystallographic orientation map (Figures 4K and 4L) reveals a strong preferential crystallographic orientation of <10-14 > axes and the c-axis, as confirmed by a map of the inverse pole figure orientation (Figures 4M and 4O). Larger crystals belonging to other crystallographic domains occur away from this region (Figures 4J–4L).

Notably, EBSD analysis reveals relics of micritic aragonite enclosed within the sparry calcite crystals, forming a poikilotopic texture (Figures 4C and 4G). Aragonite micritic cement occurs between stacked layers (Figures 4C, 4D, S4B, and S4F). There is no preferred orientation of the aragonitic relics measured in either crystallographic orientation maps or pole orientation of the a, b, and c axes (Figures 4D and 4E).

### Electron microprobe analysis

EPMA was employed to determine the concentration of targeted elements in different parts of the skeleton and in the carbonate from the host rock. Measurements at the central part of the plates and rings (Figures S10 and S11; Tables S6–S10; n = 41) reveal elevated Calcium (mean = ca. 39 wt %) and Strontium (mean = ca. 11,800 ppm or 1.18%) relative to the sclerite margins. Sr concentration is lower at the sclerite margins (mean = ca. 6,190 ppm) compared with the central portion of the sclerites. Mg, Mn, and Fe are notably concentrated at the skeleton margins relative to the inner region (means of ca. 160, 44, and 570 ppm, respectively). The carbonate from the host rock (n = 12) has the lowest Sr values (mean = ca. 2,590 ppm). Mn and Fe have means of ca. 830 and 3390 ppm, respectively. The concentration of Mn and Fe in the skeleton margins and in the host rock carbonate is consistent with the enhanced CL luminescence in these regions.

## Discussion

### A cataphract skeleton formed by controlled biomineralization

The sclerites are independent calcareous plates and rings regularly organised, usually imbricated and stacked in at least two layers (Figures 1B, 2A–2F, and S5; Video S1). Sclerites formed as repeated elements with a sigmoidal cross-section, and each layer has a regular and divergent manner of imbrication, to form a cataphract skeleton (Figures 1A, 1B, 2B, 2D, and 2E). Slight variations of sclerite shape can be explained by variability in the position/angle that sclerites created during compaction. Since sclerites articulate, closely-spaced sclerites are offset along their length and/or rotated with respect to each other to yield cross-sections with different shapes (Figure 2D-III). Plates alternate along the midline of each tube face, producing an articulating axis of plates, with imbricated rings grading into imbricated plates (Figure 2A).

Although the sclerites of *Corumbella* are now preserved as neomorphic low-Mg calcite, original aragonitic mineralogy is suggested by both elevated Sr content (relative to the host rock carbonate) and the presence of aragonitic relicts embedded in neomorphic calcite ‒ both widely accepted criteria for identifying the primary composition of carbonate skeletons.^13^^,^^14^ Multiple regions of sclerites have been analyzed yielding a limited number of preserved, nano-scaled, aragonitic relicts (Figures 4F and 4G), so accounting for the limited data points measured in EBSD that create a scattered distribution (Figure 4E).^15^ The discovery of the material with very low alteration could yield a direct measurement of more relicts, preserving a wider area of the sclerites. In the Ediacaran, preserved aragonite had been only reported to date from the microfossil *Protolagena*,^16^ while a primary aragonitic composition for *Namacalathus* and *Cloudina* has been proposed based on isotopic proxies.^17^

Uncertainties remain as to whether some Ediacaran skeletal taxa were originally biomineralized. For example, Yang et al.^18^ questioned the biomineralization of cloudinids, while Shore and Wood^19^ showed that they had a mineralized and flexible skeleton. Aragonite can precipitate as cement after the death of organisms, preserving organic remains. For example, modern siboglinids (marine polychaete annelids) can be coated by aragonite crystals after death at hydrothermal seeps,^20^ where the inorganic aragonite envelopes the organic tube replacing organic layers in the ‘rooted’, proximal part of the tubes within the sediment.^20^ A similar origin for the aragonite in *Corumbella* is not supported, however. First, calcite crystals have a consistent c-axis alignment almost perpendicular to the tube main axis. Second, preservation is via a film of framboidal pyrite pseudomorphs coating the sclerites (that may indicate a decay-prone tissue in the external part of the sclerites – see discussion below), as would not be expected from aragonite replacement/embedding rather than original skeletal material.

An alternative explanation that the bilayered wall was created by dissolution is also highly unlikely. We show 3D specimens with consistently articulated sclerites forming bilayered walls with a rough inner surface (e.g., Figures 1B, 2E, 2F, S5B, and S8D–S8F; Video S1), supporting a primary biological feature rather than a diagenetic dissolution origin for the wall layering. We also recognise a consistent architectural pattern of skeletal microstructure that is noteworthy for its organizational complexity, namely repetitive articulating imbricated elements with constant shape, stacked in layers with consistent and inverse imbrication patterns. Moreover, the presence of sediment and aragonitic cement between skeletal layers (Figures 1, 3, and S4) would not be expected to have been formed during a stage of skeleton dissolution prior to sediment lithification. In such a scenario, the cavities would be filled with spar cement, which is absent here. Finally, alteration has impacted the margins of the sclerites more severely, as demonstrated by the distinct luminescence pattern (Figure 1) coupled with EPMA showing a decrease of Sr content and an increase of Mn content toward the edges of each sclerite (Table S10).

We note a preferential co-alignment of the c-axis of the calcite crystals within the sclerites almost perpendicular to the tube length (Figures 4K and 4L). The transitional change of crystallographic orientation of the c-axis (Figure 4L) from right to left in the crystallographic orientation map (Figure 4K) might reflect the variation in sample sectioning direction. Large crystals closer to the more altered sclerite margin possess more random crystallographic orientations (Figure 4K, upper left corner). Since diagenetic recrystallization commonly yields randomly orientated crystals,^21^ the large crystals may reflect stronger recrystallization. It is likely that the original crystallographic orientation has been preserved in less altered regions (see Data S1 for an appraisal of the orientation patterns of other taxa). Original textures can be finely preserved during neomorphism since organic material can be partially degraded, permitting percolating solutions to precipitate calcite within voids.^22^ Positive feedback of dissolution of aragonite readily followed by local supersaturation and precipitation of calcite produces pseudomorphs with inherited crystallography after epitaxial growth.^22^^,^^23^ It has been proposed that acicular carbonates can be replaced by fibrous calcite that retains the original c-axis orientations.^24^ When porosity is reduced, e.g., by locally preserved organic material,^25^ the dissolution-reprecipitation front is inhibited,^23^ and aragonite relicts are preserved. Poles are organized in subgroupings expressed in a slightly spread pattern (Figure 4L), thus indicating a subtle variability in orientation. This might have been created by the fact that sample sectioning is not always exactly transverse to the sclerite. We can rule out the effect of tectonic deformation since each subgrouping is comprised of plots of several crystals and the crystallographic pattern is not homogeneous across the analyzed area (compare left upper corner with center of Figure 4K), so there is no consistent orientation parallel to dominant stress fields. Conversely, we would expect that each deformed crystal would have its own subgrouping with a spreading pattern, but each crystal has a consistent orientation pattern (Figure 4K).

A composite calcareous-organic skeleton is suggested by the Raman data. Organic tissue embedded within the calcareous skeleton would be recalcitrant and yield amorphous carbon^26^^,^^27^ (Figure 3B). An organic template^28^ into which the sclerites were embedded can be inferred by the presence of oxidized pyrite framboids now present as Fe-(oxyhydr)oxides (Figure 3). Pyrite framboids are known to form preferentially and rapidly during very early diagenesis associated with decaying labile organic matter.^27^ The presence of soft tissue attached to the skeletal plates might account for the integrity of the cataphract skeleton, the decay of which would account for commonly disarticulated specimens (Figures 2G and S8B). Compaction has yielded some fragmented specimens (Figures 1F and 1G).

The replacive, diagenetic calcite likely mimics an originally laminar organo-aragonitic microstructure of the *Corumbella* skeleton. Orientated crystals can form when several crystals nucleate, grow preferentially along the c-axis, and then coalesce,^14^ as might be expected for the original aragonitic microstructure of *Corumbella*. It is possible that organic envelopes and surfaces enable the growth of aragonite crystals with consistent c-axis alignments, given that organic material controls biomineralization by providing confined volumes, in which ionic conditions favor aragonite precipitation over calcite.^28^^,^^29^^,^^30^^,^^31^ In *Corumbella*, the crystallographic pattern is similar in different crystals both above and below a zone with voids (Figure 4K), and is laterally continuous in neighboring sclerites which may represent an organic-rich layer situated at the contact between two mineralized layers (Figure S4B). Continuity of crystallographic pattern across skeletal layers occurs in calcitic and aragonitic microfabrics.^32^ The co-alignment of <0001> and <10-14 > axes suggests a highly ordered sheet structuring.^33^ We conclude that *Corumbella* skeleton is now formed by replacive sparry calcite crystals of a probably original laminar microstructure of aragonite. This type of layered skeletal carbonate microfabric has a stepped process of formation that is biologically controlled.^29^^,^^34^ Mineralized layers are progressively stacked upon previously deposited layers. This process (the so-called ‘common strategy’ for carbonate layered skeletal microfabrics) is controlled by the mineralizing ectodermal epithelium, where organic material delimits the resulting layer and the shape of the organic envelope molds the crystal shape.^29^ Even if it is assumed that the pyritic film along the sclerites margin represents an organic layer coating the sclerites (template for biomineralization) that are derived from the epidermis (as similarly proposed for chancelloriids^35^), it is not possible to infer the position of sclerites relative to the epidermis itself.

The overall morphology of *Corumbella* shows a directional polarity,^6^ and this is also manifested by the consistent directional imbrication of the plates and rings (Figures 1A, 2A, 2B, 2E, and S8F) and the presence of a possible set of thick rings at the tube base (Figures S4A, S4B, and S8F). This polarity has been interpreted as oral-aboral,^6^ but is here changed to ‘proximal-distal’ as we question the affinity of *Corumbella* to Cnidaria (see below; Table S1). Two orthogonal planes across the midlines divide the skeleton into four quadrants, yielding 4-fold radial symmetry^5^^,^^6^ (Figure 2C). The consistent shape and organization of the sclerites, as well as regular microfabric and consistent crystal orientation^28^ shows that considerable biological control was exerted over crystal growth and skeletal formation in *Corumbella.*

### Appraisal of *Corumbella* biological affinities

It has been proposed that *Corumbella* could be related to scyphozoan conulariids based on the 4-fold radial symmetry (related to polygonal cross-section) plus the presence of lateral edges (corners in conulariids), and midlines^3^^,^^4^^,^^6^^,^^8^^,^^9^ (see Table S2 for a summarized comparison). As for the alternation of plates at either sides of midline in *Corumbella*, conulariids have an alternation of skeletal features (transverse ribs) along either side of the midline (e.g., *Climacoconus*^7^). We consider the tube polygonal cross-section of *Corumbella* and the midlines as valid morphologies, *contra* Walde et al.^10^ (see Data S1).

However, while conulariid tubes have a polygonal cross-section, *Corumbella* tube grades from a uniseriate proximal region with an almost circular cross-section, formed by rings, to a distal region with polygonal cross-section and 4-fold symmetry, formed by plates. The Ordovician conulariid *Metaconularia anomala* has a variation of faces number along the periderm,^36^ but neither a variation of structures occurs nor is there a change from a circular to a polygonal cross-section, as found in *Corumbella*. The polygonal cross-section (and, consequently, the associated squarish corner in conulariids or lateral edge of *Corumbella*) is not exclusive to either conulariids or cnidarians (e.g., Hydrozoa). For instance, polygonal tubes are also shared by some annelid serpulids^37^ and by *Sinotubulites*.^38^^,^^39^ Additionally, radial (4-fold) symmetry neither unequivocally relates *Corumbella* to conulariids, nor places this genus within the Cnidaria.

Bilateral symmetry could have evolved earlier than the bilaterian common ancestor^40^ since polyps of anthozoan cnidarians, for instance, have biradial and/or bilateral symmetry.^41^^,^^42^^,^^43^ Moreover, some possible bilaterians are known to have radial symmetry (e.g., cycloneuralians and echinoderms), including the terminal Ediacaran *Namacalathus*.^44^ The type of symmetry, either radial or bilateral, has been associated with sessile and vagile habits, respectively, and so might be only a weak trait for phylogenetic reconstitutions.^45^ The radial symmetry of *Corumbella* is consistent with a sessile lifestyle.

Conulariids have a single-unit skeleton (periderm) and thus midlines are regions of continuous skeleton. The periderm was organic or organo-phosphatic and has a generally consistent internal layering (macro- and microlamellae) among transverse ribs and the wall is smooth internally.^46^ The periderm has internal carinae formed by thickening. Cambrian phosphatic and soft-bodied conulariid-related taxa (*Olivooides* and carinachitids) are double-layered, with continuous walls that are smooth internally.^47^^,^^48^ The lamellar nature of *Corumbella* skeleton was used to support a conulariid affinity,^6^ but these lamellae have not been ruled out as platy calcite that now builds the skeleton, or host rock aluminosilicate crystals parallel to the skeleton wall surface. Their scale is also different from the lamellae in conulariids.^46^ *Corumbella* likely possessed a laminar microfabric, but this feature is not exclusive to conulariids since it is also found in possible cnidarians and bilaterians, for example.^44^ It has been interpreted that plates with pores and papillae would form the skeleton of *Corumbella*, as in conulariids,^9^ but these features could be calcite dissolution artifacts.^10^ In contrast to previous work,^5^^,^^6^ we think that a conulariid affinity to *Corumbella* is now unlikely, as we show that (1) *Corumbella* has an inferred original aragonitic skeletal composition, (2) the skeleton is multi-element, formed by independent articulating isolated or more commonly imbricated sclerites forming two layers (i.e., cataphract skeleton), with open midlines as being articulating axes (Figure 2; Table S1), (3) internal lamination is restricted to single sclerites, (4) the skeleton has a rough internal surface, (5) the skeleton is devoid of any internal thickening, and (6) there is a more gradual variation in the width of the *Corumbella* tube compared to the abrupt changes seen in conulariid periderm.^4^ While it has been suggested that articulated annular elements could form the *Corumbella* skeleton,^49^ it is not clear whether the authors consider such rings as being independent elements; most likely these were interpreted as being continuous.^49^

The cataphract organization of *Corumbella* is also not consistent with extant and fossil cnidarians (see Table S2). The morphological variation along the tube (uniseriate proximal region with rings grading to quadriseriate distal region with plates), a bilayered wall with regular and inverse imbrication mode of the sclerites in each layer, a multi-element articulated imbricated skeleton, and an alternation of independent, articulated plates along the midlines (articulation axes) is not consistent with a cnidarian affinity. Staurozoans, cubozoans, and living scyphozoans are unmineralized^50^ (including Cambrian taxa^51^). Although *Corumbella* shares an aragonitic composition with hydrozoans,^34^^,^^52^ the latter have a massive skeleton,^34^ in opposition to the multi-element skeleton of *Corumbella*. *Corumbella* composition and microstructure also differ from the Cambrian *Sphenothallus*.^53^ Although several anthozoans have a multi-element skeleton of sclerites/spicules, some have a central axis in the skeleton.^54^ Polyps of the subclass Ceriantharia,^55^ probably one of the oldest extant lineages of Cnidaria,^43^ form tubes that are constructed by independent, overlapping rings built by filaments of ptychocysts (with a protein matrix^56^), but the absence of more detailed studies on the composition of the tube in Ceriantharia limits further discussion^42^^,^^55^ (Table S2). The earliest probable octocorals are from the Cambrian.^57^^,^^58^ For example, *Echmatocrinus brachiatus*^57^ possessed sclerites, but their morphology, arrangement, and composition are different from *Corumbella*. Other Cambrian anthozoan candidates are unmineralized or possess internal thickenings.^7^ Ediacaran putative anthozoans possess internal cross-walls and are possibly unmineralized,^7^ thus differing from *Corumbella*. Neither the massive skeleton of both scleractinians and blue corals^4^ occurs in *Corumbella*. The platelets, scales, and shingles^56^^,^^59^^,^^60^^,^^61^ of some cnidarians are only surficial, and do not build an articulated bilayered skeleton as in *Corumbella*. Since the original habit of aragonite crystals from *Corumbella* skeleton is not currently known, any further comparison with skeletal microfabrics of cnidarians is not possible.

Alternatively, it has been proposed that *Corumbella* was a sinotubulitid or a sabelliditid polychaete^10^ (see Table S3). Sinotubulitids possessed external irregular, occasionally bifurcating ornamentations (‘ribs’) (Figures 9A–9E of Walde et al.^10^; Figure 3B of Chen et al.^38^) and had a continuous wall with smooth interior (Figures 7C–7G of Walde et al.^10^). Some 3D specimens with likely circular cross-sections and more regular annulations shown by Walde et al.^10^ (e.g., their Figure 7A) could be proximal parts of *Corumbella* (e.g., Figures S1B, S3B, S4, S8C, and S8D) or, alternatively, a different taxon. Sabelliditids have a one-piece tube with continuous wrinkles in the longitudinal section.^62^ But by contrast, *Corumbella* possessed sclerites with a regular non-bifurcating external pattern (Table S3; Figures S1A and S3A), and non-continuous wall with rough inner surface (Figure 1B). A polychaete annelid affinity is unlikely since the cataphract skeletal architecture of *Corumbella* is not found in polychaetes, and siboglinids are unmineralized^20^^,^^63^ (see Data S1 and Table S4 for further comparison).

*Corumbella* has also been compared to the problematic organic and flexible tubular Cambrian fossil *Onuphionella*, and to the putative annelid *Eoalvinellodes annulatus*^10^, but this is not supported by morphological and compositional evidence. *Onuphionella* skeleton purportedly agglutinated muscovite flakes,^64^ and *Eoalvinellodes annulatus* ornamentation is irregular, sometimes bifurcating and there is no evidence for tube’s flexibility (Figures 18A–18C of Georgieva et al.^65^), which are in marked contrast to the regularity and non-bifurcating morphology of *Corumbella* plates and rings (Figure S3), and to *Corumbella* tube inferred flexibility (Figure S8A).

Another tubular Ediacaran fossil made up of independent elements is *Costatubus*, that is a late Ediacaran tubular fossil found in the Wood Canyon and Deep Spring formations, USA.^66^ However, this genus differs from *Corumbella*, as *Costatubus* has a single wall, with smooth lumen. The building elements of *Costatubus* only slightly overlap, are continuous, barrel-shaped, with a seemingly more constant thickness relative to *Corumbella* sclerites in tube longitudinal section (not a sigmoidal shape as in *Corumbella*). Additionally, the tube has circular cross-section.

A cataphract organization creating a scleritome is established for articulating small shelly fossils (SSFs), such as mineralized plates, shells, and spines of bilaterians and still enigmatic taxa, which appeared in the latest Ediacaran.^67^^,^^68^^,^^69^ Small carbonaceous fossils (SCFs) (ecdysozoan-like organic sclerites^70^) are also known from the terminal Ediacaran. The earliest complete cataphract skeletons (scleritomes) known have been derived from exceptional preservation in early Cambrian strata.^71^^,^^72^ In the Ediacaran-Cambrian transition (including the Cambrian Stage 2), SSFs are dominantly assigned to lophotrochozoans,^68^^,^^73^ such as the tommotiids,^72^ molluscan halkieriids,^71^ and putative lophotrochozoan cambroclavids,^73^^,^^74^ as well as probably to Chaetognatha and other taxa with undetermined affinities^73^ (see Table S11). It was only later in the Cambrian Stage 3 that biomineralized ecdysozoans and deuterostomes radiated.^73^ Machaeridians (annelids that appeared in the early Ordovician) also possessed a cataphract skeleton.^75^^,^^76^

Biomineralized skeletal elements are also found in taxa with more basal metazoan affinities. Cambrian chancelloriids, which have been long interpreted as poriferans, possessed a soft body with external sclerites, but these did not form a *sensu strictu* cataphract organization, that is imbricated, articulating elements. Recently, it has been shown that chancelloriids sclerites had an outer organic layer, that was interpreted as an organic template (derived from the epidermal integument) for biomineralization of the internal, aragonitic, fibrous layer.^35^ This evidence points to a eumetazoan (and therefore epitheliozoan), rather than poriferan affinity.^35^ Our interpretation that *Corumbella* sclerites also possessed an outer organic template would support the placement of this taxon within the Eumetazoa.

The possibly laminar aragonitic microfabric of *Corumbella* is reminiscent of carbonate laminar microfabrics of lophotrochozoan skeletons, such as in bryozoans and molluscs.^29^^,^^33^^,^^77^ However, laminar microfabrics are also found in other metazoans, like cnidarians. Indeed, laminar aragonitic skeletal microfabric appears to be shared by cnidarians and bilaterians.^44^

*Corumbella* is the oldest metazoan described to date with a complex cataphract skeleton with biologically-controlled biomineralization and may represent, along with potentially *Namacalathus*^44^^,^^78^ and possibly *Cloudina*,^79^ the earliest fauna of biomineralized bilaterians.

Although the cataphract organization is reminiscent of Cambrian bilaterians, unequivocal evidence for a bilaterian affinity has not been found to date in *Corumbella*. Although we provide a detailed morphological reconstruction for *Corumbella*, it is crucial at least to infer hypothetical ancestors through a phylogenetic study before any evolutionary interpretations of body plans can be considered.^80^ Morphological analyses of Ediacaran metazoans often fail to accommodate them within modern phyla owing to their basal characters, which can often be similar or shared with basal groups of other phyla, as well as to the lack of soft-tissue preservation.^81^^,^^82^ Thus, most Ediacaran and Cambrian eumetazoans can often only be assigned to total groups.^82^ For example, basal bilaterians would have had features conserved from Eumetazoa, unique characters, as well as some characters shared by the extant members of the phyla.^82^^,^^83^ The morphogenetic diversification of bilaterian phyla body plans might occur only in the Phanerozoic, with the evolution of crown groups.^82^ *Corumbella* features conform to this rationale for basal eumetazoans, as it possessed features usually attributed to cnidarians (polygonal cross-section and radial symmetry), as well as a cataphract skeleton, as found in some bilaterian and problematic, Cambrian taxa.

### Biologically controlled biomineralization and cataphract skeletons: Ecological implications

*Corumbella* possessed strongly biologically controlled calcareous biomineralization that would require a genetic control for the development of: (1) repeating sclerites, formed possibly by vertical accretion of orientated organic-rich aragonite crystals embedded within an organic-matrix, (2) changing of morphology from an uniseriate region formed by continuous rings to a region with repeated increments of four articulating plates each, (3) controlled sclerite shape, (4) sclerite growth cessation allowing articulation and alternation at the midlines, (5) sclerite imbrication consistently inverse in different layers, and (6) a fixed number of skeletal layers (Figures 2A–2F). This level of skeletal organization, and thus biological control, is not seen in either extinct (including Ediacaran and Cambrian cnidariomorphs summarized by Van Iten et al.^7^ and Kouchinsky et al.^73^) or living cnidarians (Table S2), nor other known Ediacaran skeletal metazoans^38^^,^^84^^,^^85^^,^^86^ (Table S3).

It has been considered that biomineralizers expanded to potentially low carbonate-saturation clastic settings only in the Cambrian, with the development of evolutionary constraints for biomineralization.^87^ We hypothesize that the occurrence of *Corumbella* in mixed siliciclastic-carbonate settings, and in predominantly clastic beds thus also supports a strong control of biomineralization that enabled the colonization of siliciclastic settings in the latest Ediacaran. This proposal is consistent with the proposal that the ‘Cambrian radiation’ was a transitional event with roots in the Ediacaran.^68^^,^^88^

*Corumbella* had a complex skeleton, relative to other Ediacaran biomineralizers, that implies a strong biological control over biomineralization. *Corumbella* serves a datum (in a growing line of evidence) that implies the Cambrian radiation of bilaterians may have had deep roots.^78^^,^^79^ If true, we might also expect that flexibility (Figure S8A), efficient support, protection against predation, feeding efficiency, and motility in biomineralizing taxa (behaviors that are correlated with biomineralization and biomechanics, particularly of multi-element skeletons) to have emerged by the end of the Ediacaran Period. Future work, in the form of new fossil discoveries or computational analyses, may test these hypotheses.

### Limitations of the study

Previous work has helped to elucidate the morphology of *Corumbella*,^6^ and here we develop this further with the finding of a cataphract skeleton reminiscent of early Cambrian SSFs, which comprise both bilaterians and problematic taxa. But establishing the affinity of *Corumbella* is still challenging. This limitation is common for late Ediacaran mineralized taxa and is best resolved when soft tissues are found in association with skeletal remains.^78^^,^^79^ Our detailed morphological reconstruction with potential new findings of soft tissues could yield new insights into the affinities of *Corumbella*.

## STAR★Methods

### Key resources table

**Table:** 

REAGENT or RESOURCE	SOURCE	IDENTIFIER
**Deposited data**		

RAW micro-CT data	This study	Mendeley Data (https://doi.org/10.17632/y2rb6sfvcz.1)
RAW micro-Raman spectroscopy data	This study	Mendeley Data (https://doi.org/10.17632/y2rb6sfvcz.1)
RAW EBSD data	This study	Mendeley Data (https://doi.org/10.17632/y2rb6sfvcz.1)

**Software and algorithms**		

Renishaw WiRE™ 4.1	Renishaw	https://www.renishaw.com/en/raman-software--9450
Oxford Instruments AZtecSynergy	Oxford Instruments	https://nano.oxinst.com/products/aztec/aztecsynergy
Oxford Instruments HKL Channel5	Oxford Instruments	https://nano.oxinst.com/products/aztec/aztechkl
VGstudio Max 2.1	Volume Graphics	https://www.volumegraphics.com/en/products/vgsm.html
ImageJ 1.46r	National Institutes of Health, USA	https://imagej.nih.gov/ij/
Microsoft Excel	Microsoft Corporation	https://www.microsoft.com/en-us/microsoft-365/microsoft-office

### Resource availability

#### Lead contact

Further information and requests for additional data should be directed to and will be fulfilled by the lead contact, Mírian Pacheco (forancelli@ufscar.br).

#### Materials availability

This study did not generate new unique reagents.

### Experimental model and subject details

Samples are housed in the Palaeontological Collections of the Laboratório de Paleobiologia e Astrobiologia, Universidade Federal de São Carlos, Sorocaba, Brazil (Samples: CAP/1A 1020, which corresponds to CAP/1F 11; CAP/1F 12; CAP/1A 1022; CAP/1A 1023; CAP/1A 1024; CAP/1A 1025; CAP/1A 1026; CAP/1A 1071; CAP/1F 14), of the Instituto de Geociências, Universidade de São Paulo, São Paulo, Brazil (Samples: GP/1E 4182, GP/1E 574a, GP/1E 4210, GP/1E 5186 and GP/1E 5819), and of the Departamento Nacional de Produção Mineral (DNPM), currently Agência Nacional de Mineração (ANM), Rio de Janeiro, Brazil (Sample DGM-5601-I).

### Method details

#### Cathodoluminescence

Luminescence was imaged using highly polished thin sections and embedded blocks in resin using a Cathodoluminescence Cold Cathode CITL 8200 MK3A coupled with a Nikon microscope in the School of GeoSciences, University of Edinburgh. We used a x10 objective lens, current of 900-1000 A and voltage of 25-30 V.

#### Raman spectroscopy

Thin sections were imaged using Raman micro-spectroscopy at the Bioimaging Facilities, School of Engineering, University of Edinburgh. We used a Renishaw micro-Raman system mounted on optical microscope and laser source of 785 nm excitation line. For map acquisition, equipment was calibrated by the Si band (520.7 cm^-1^), then measurements were performed with a x20 magnification objective lens, with an exposure time of 10 s, and laser power of 10%, between 300-1700 Raman shift/cm^−1^. We processed data in the software Renishaw WiRE^TM^ 4.1.

#### Electron microscopy

Backscattered electron (BSE) imaging and energy-dispersive X-ray spectroscopy (EDS) analyses were made using a carbon coated highly polished embedded block (20 kV voltage and 6.8 and 6.9 mm working distances). Later, this sample was further polished and recoated for electron backscatter diffraction (EBSD). We used 15 kV voltage, 3.0-3.5 nA current, a 70° pre-tilted holder and a Hough Resolution of 100, 9 bands (edges) and indexing for both calcite and aragonite phases. We acquired data with Oxford Instruments AZtecSynergy software, and later processed data in the Oxford Instruments HKL Channel5 software with Tango and Mambo modules. BSE, EDS and EBSD analyses were performed in a Carl Zeiss SIGMA HD VP Field Emission SEM, an Oxford AZtec ED X-ray analysis, and an Oxford AztecHKL EBSD system at the School of GeoSciences, University of Edinburgh.

#### Electron probe micro-analysis

A highly polished thin section and an embedded block, both carbon coated, were analysed using electron probe micro-analysis (EPMA) in a Cameca SX100 instrument at the School of GeoSciences, University of Edinburgh. We used a beam size of 3 μm, voltage of 5 kV, and currents of 2 nA and 60 nA. A highly polished thin section was carbon coated and analysed using EPMA in a JEOL JXA-8530F instrument at the Institute of Geosciences, University of São Paulo. We used beam sizes of 2 μm, 2.5 μm, 3 μm and 5 μm, voltage of 15 kV, and current of ca. 2E-008 A.

#### Micro-computed tomography (micro-CT)

Micro-Computed Tomography (micro-CT) measurements were carried out by means of synchrotron radiation at the former beamline W2 for materials science at the storage ring DORIS (DESY, Hamburg, Germany). The micro-CT data of the specimen were collected using a beam-energy of 35 keV and a lens coupled CCD camera built of 3056x3056 pixels with a physical pixel size of 12 μm. An optical magnification was given by modifying the position of the lens (Nikkor; Nikon GmbH, Nikon Professional Services, Tiefenbroicher Weg, Düsseldorf) between the scintillator (CdWO4, with a 450 μm thickness) and the CCD camera. An effective pixel size of 3.25 μm was achieved in the recorded images for the specimen to fit in the field of view. Data processing has been made using the softwares VGstudio Max and ImageJ.

### Quantification and statistical analysis

Statistical analyses of electron probe micro-analysis were made in the software Microsoft Excel by determining minimum, maximum, mean and standard deviation values. Statistical details of experiments can be found in the results section of the main text and in Table S10 of the supplemental information.

## Data Availability

• RAW data from micro-CT, Raman spectroscopy and EBSD analyses have been deposited at Mendeley Data (https://doi.org/10.17632/y2rb6sfvcz.1) and are publicly available as of the date of publication. Accession numbers (DOI) are listed in the key resources table. • This paper does not report original code. • Any additional information required to reanalyse the data reported in this paper is available from the lead contact upon request.

## Supplemental information

Document S1. Figures S1–S11 and Tables S3–S10

Table S1. Comparison of morphological features and terminology published for *Corumbella* and proposed for this study. Table based on Table 1 from Pacheco et al.^1^, related to Figures 1, 2, 3, and 4

Table S2. Comparison of *Corumbella* and cnidarian features, related to Figures 1, 2, 3, and 4

Table S11. Comparison of features of *Corumbella* and of living and extinct bilaterians and extinct possible bilaterians, related to Figures 1, 2, 3, and 4
