# Supplementary material for: Ediacaran Corumbella has a cataphract calcareous skeleton with controlled biomineralization
Source: iScience. 2022 Nov 25;25(12):105676. doi: 10.1016/j.isci.2022.105676 (PMC9763863; doi:10.1016/j.isci.2022.105676)
Supplement: Document S1. Figures S1–S11 and Tables S3–S10 [file mmc1.pdf]

iScience, Volume *25*

Supplemental information

Ediacaran *Corumbella* has a cataphract calcareous skeleton with controlled biomineralization

Gabriel Ladeira Osés, Rachel Wood, Guilherme Raffaeli Romero, Gustavo Marcondes Evangelista Martins Prado, Pidassa Bidola, Julia Herzen, Franz Pfeiffer, Sérgio Nascimento Stampar, and Mírian Liza Alves Forancelli Pacheco

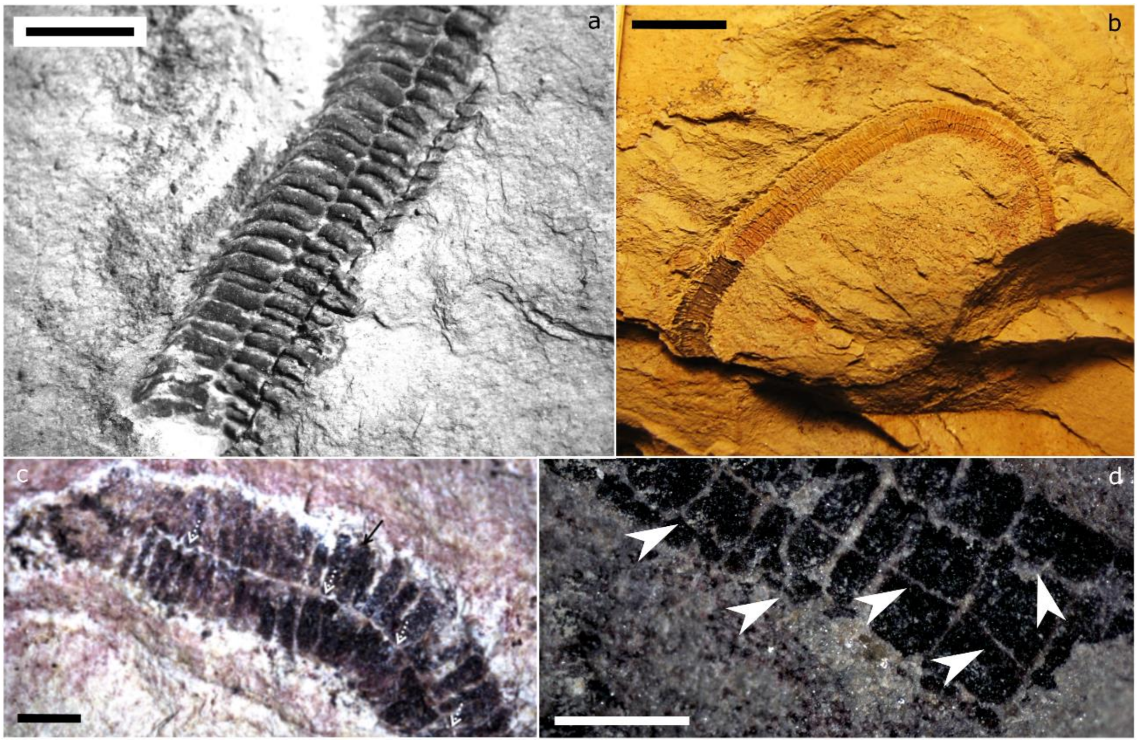

Figure S1. Midline and taphonomic breakages in *Corumbella werneri*, related to Fig. 1. Samples GP/1E 4210 (A), DGM-5601-I (B), GP/1E 5186 (C) and GP/1E 5819 (D). A: Tube with midline, depicting the zigzagged pattern yielded by the alternate organisation of plates. B: "J" shaped bended tube with rings grading to plates. C: Folded tube, with white arrows indicating the midline and black arrow showing the lateral edge. The midline follows bending in B and C, which would not be expected for taphonomic breakages. D: Taphonomic breakages (arrowheads) cutting the plates. Scale bars: A = 2 mm; B-D = 1 mm. A and B were modified, respectively from Figs. 5E and 8C from Pacheco et al.[1] (DOI:10.1371/journal. pone.0114219), originally published under a Creative Commons Attribution License. C and D: Taken by Mírian Pacheco. Original figures have been cropped to remove excess of empty space around the fossils.

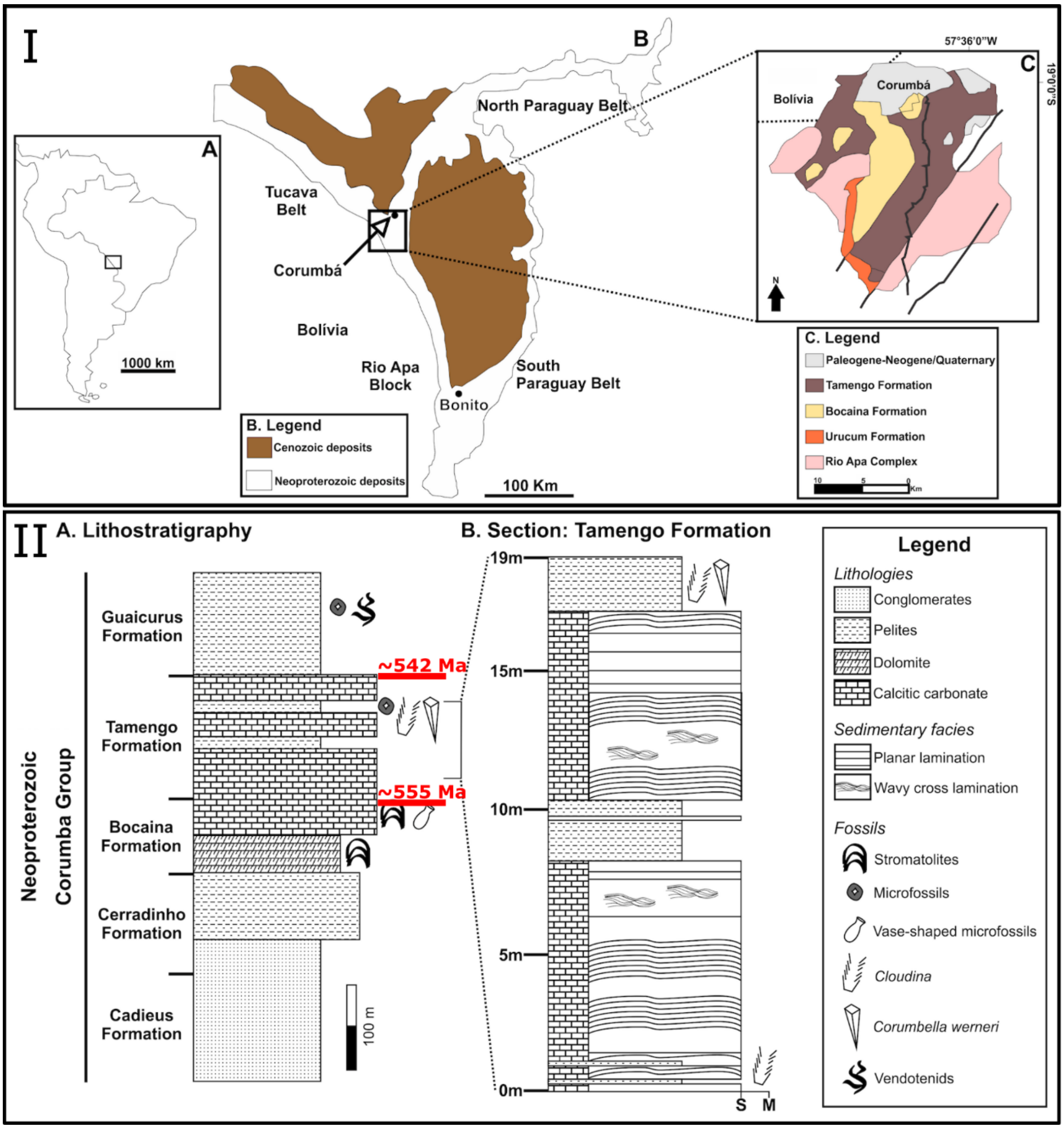

Figure S2. Geological context of the occurrence area of *Corumbella werneri* in Brazil, related to Figs. 1-4. I: Location of the Paraguay Belt in the Brazilian territory (A), geological map of the Paraguay Belt with southern region detailed (B), and distribution of geological units in the region highlighted in B (C). II: Lithostratigraphy of the Corumbá Group (A) and simplified stratigraphic section of the Tamengo Formation (B). Dates in II-A are from Parry et al.[2]. Fossils used in this study were collected in the Porto Sobramil Quarry (19°00'03.5"S, 57°37'11.7"W), Tamengo Formation. However, it is not possible to place this outcrop in the exact position within the Tamengo Formation due to the lack of stratigraphic correlation markers. I and II were modified, respectively from Figs. 1 and 2 of Pacheco et al.[1] (DOI:10.1371/journal. pone.0114219), originally published under a Creative Commons Attribution License. Fig. I was originally published by Pacheco et al.[1] with modifications after Oliveira[3]. Fig. II was originally published by Pacheco et al.[1] with modifications after Morais[4].

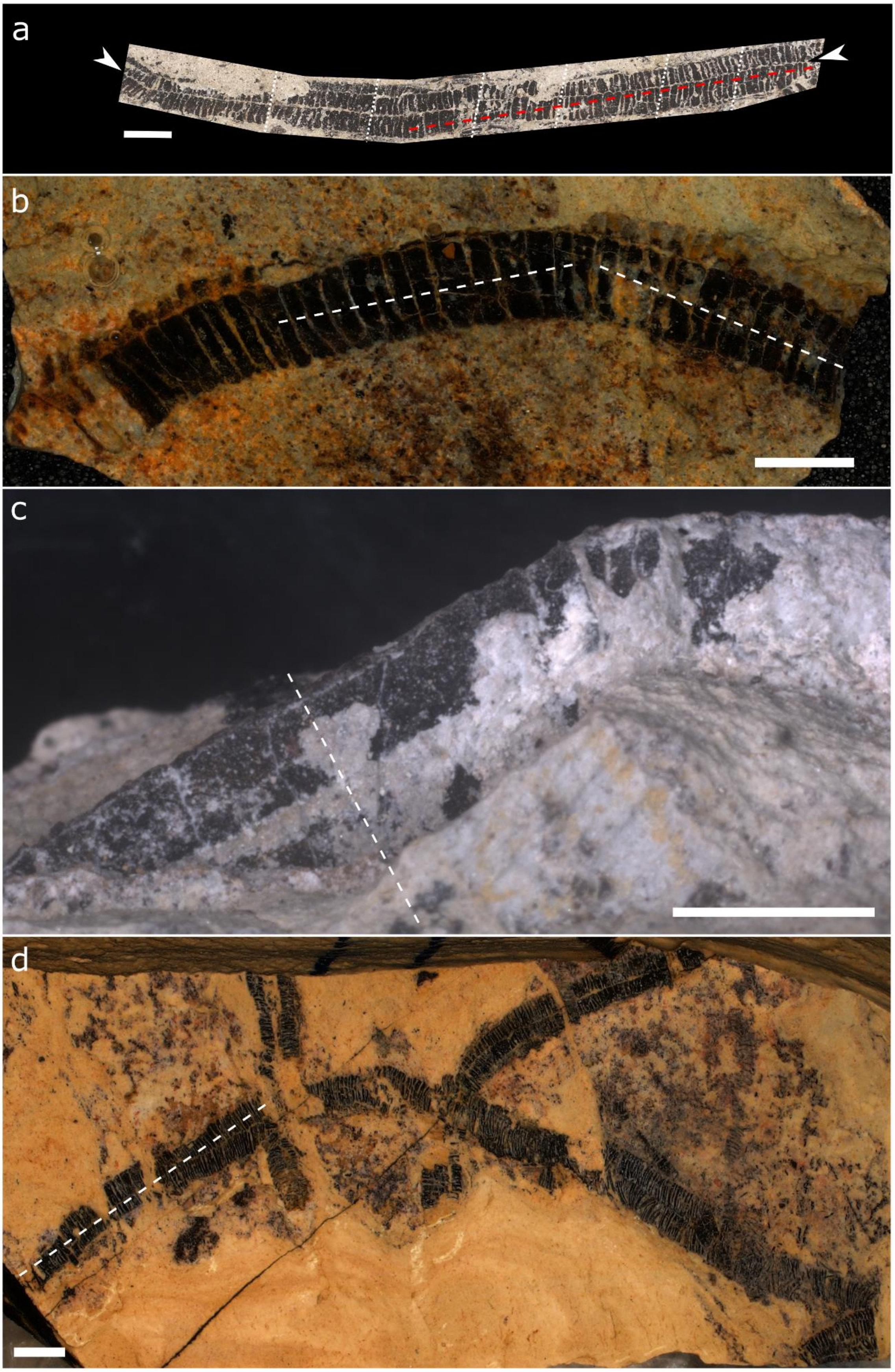

Figure S3. Samples CAP/1A 1020 (A), CAP/1A 1022 (B), CAP/1A 1021 (C) and CAP/1A 1071 (D), related to Figs. 1 and 3. A: Polyhedral part of specimen with midline and plates. The

dashed lines indicate the assembling of original images. Arrowheads point to the midline, along which convergent, non-continuous plates alternate. B: Proximal circular region of specimen with rings. C: 3D *Corumbella* specimen. D: Polyhedral part of specimens with midline and plates. A-D: Dashed line (red in A; white in other figures) indicates the cutting direction that yielded, respectively thin section CAP/1F 11 (Figs. 1A-E), polished section CAP/1A 1022 (Fig. 3C; Fig. S4), and thin sections CAP/1F 12 (Figs. 1F and G) and CAP/1F 14 (Fig. 3A). Scale bars: A-C = 2 mm and D = 4 mm. Original figures have been cropped to remove excess of empty space around the fossils and also to enable stitching (A).

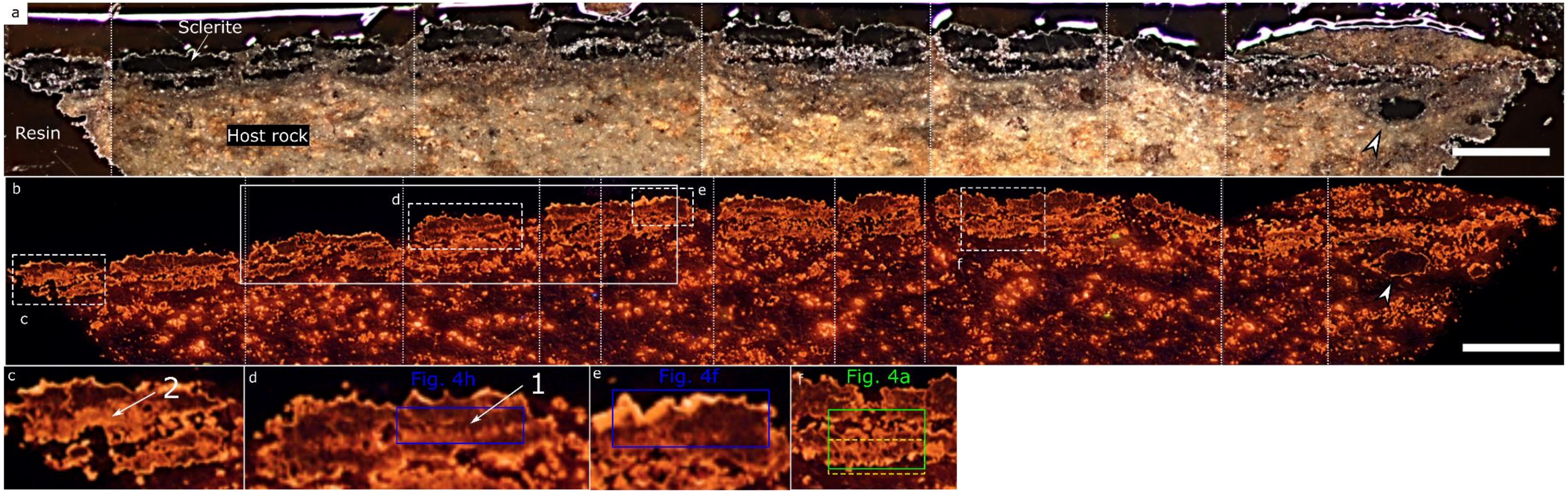

Figure S4. Sample CAP/1A 1022 (Fig. S3B), related to Figs. 3 and 4. A: Longitudinal section of the tube. B: CL image of A. White rectangle – Area of Fig. 3C. The dashed lines in A and B indicate the assembling of original images. Arrowheads point to thick elements described in the main text. C: Blotchy luminescent area (arrow 2). D: The central part of individual sclerites (rings) may show a more continuous brightly luminescent layer (arrow 1). Area analysed in Fig. 4H is delimited. E: Delimited area was analysed in Fig. 4F. F: Green rectangle – Aragonite micritic cement between stacked layers in area shown in Fig. 4A; yellow dashed rectangle -– Vertically elongated sparry crystals with palisade texture and pointy terminations that yield the irregular sclerite (ring) surface. Scale bars: 0.2 mm. Original figures have been cropped to remove excess of empty space around the fossils and also to enable stitching (A and B).

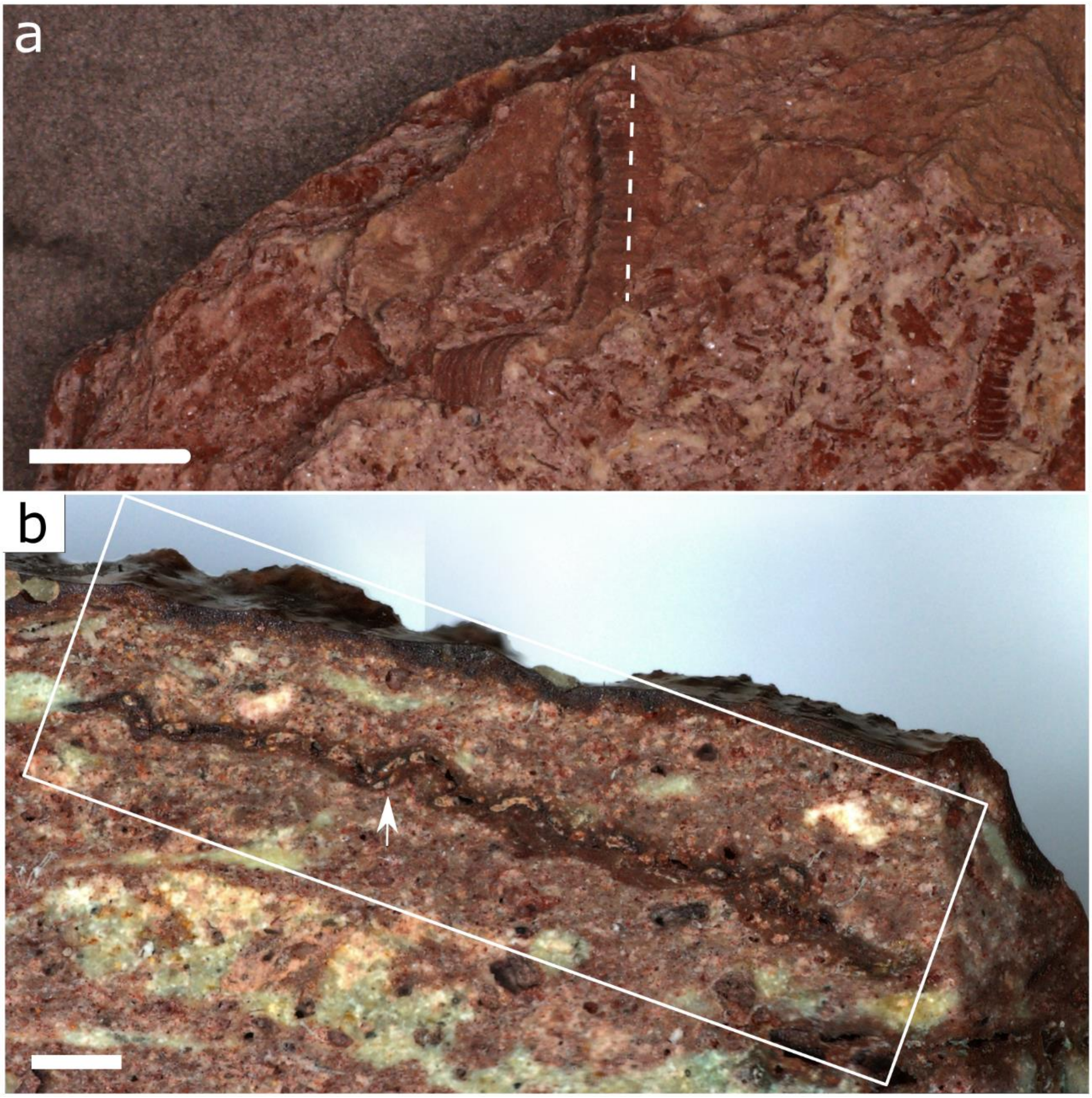

Figure S5. Sample GP/1E 4182, related to Figs. 1 and 2. A: Fossils with regions with circular and polyhedral cross-sections preserved. Dashed line indicates cutting direction that yielded polished section of B. B: Longitudinal section of polyhedral fossil in A. The rectangle highlights a single wall preserved within host sediment. The arrow points to a preserved plate that indicates that the wall is bilayered. Scale bars: A = 5 mm; B = 0.5 mm. Original figures have been cropped to remove excess of empty space around the fossils.

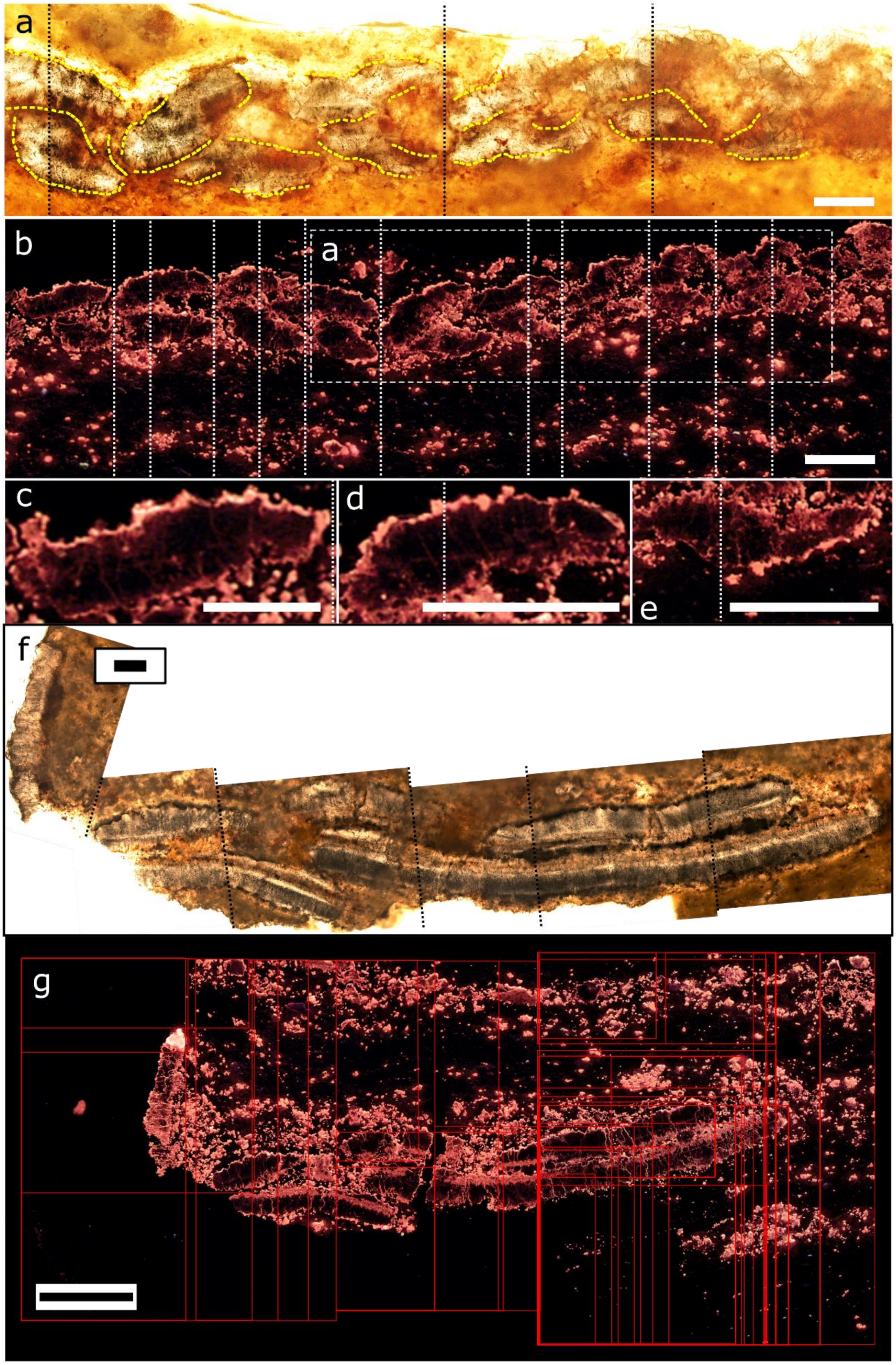

Figure S6. Identification of image assembling, related to Fig. 1. Dashed lines (A-F) and solid lines (G) indicate the margins of assembled figures. See Fig. 1 for full description. Scale bars: A,

C and F = 0.1 mm; B, D and E = 0.2 mm; G = 0.5 mm. Original figures have been cropped to remove excess of empty space around the fossils and also to enable stitching.

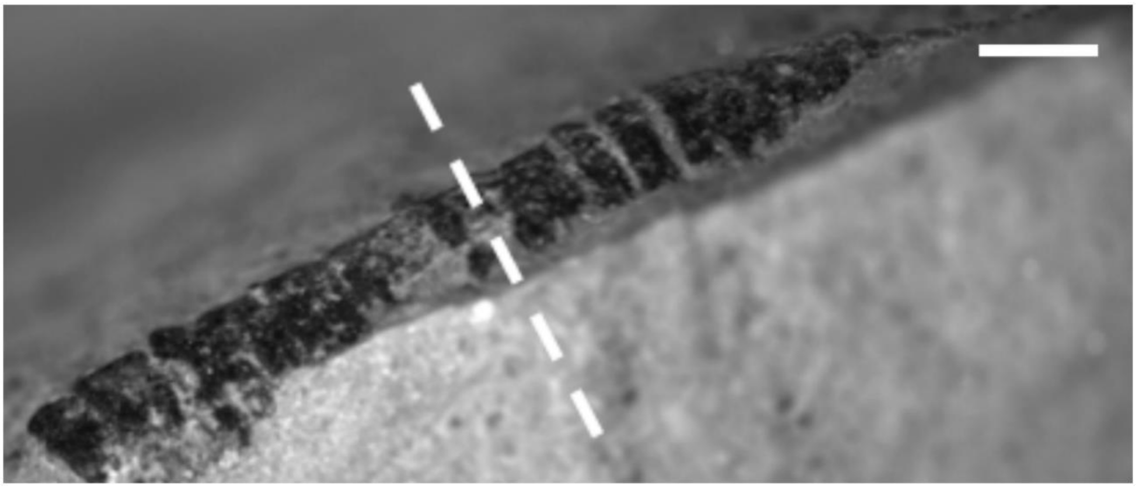

Figure S7. 3D specimen GP/1E 574a, related to Fig. 2. Dashed line indicates direction of micro-CT sectioning (shown in Figs. 2E and F). Scale bar = 1 mm. Modified from Fig. 6A of Pacheco et al.[1] (DOI:10.1371/journal. pone.0114219), originally published under a Creative Commons Attribution License. Original figure has been cropped to remove excess of empty space around the fossil.

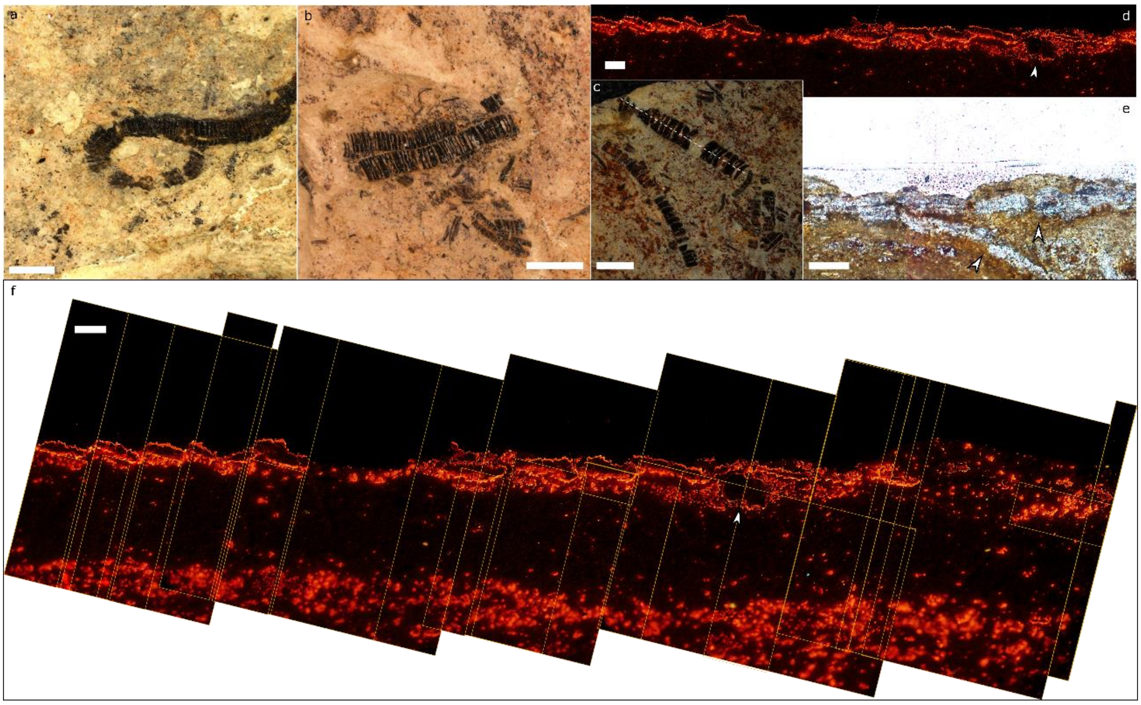

Figure S8. Disarticulation of *Corumbella*, related to Fig. 2. Samples: CAP/1A 1024 (A), CAP/1A 1025 (B), CAP/1A 1023 (C, D and F), CAP/1F 14 (E). See cartoon of types of disarticulation in Fig. 2G. A: Evidence for flexibility with sclerite articulation at the curvature. B-E: Specimens with different stages of disarticulation: 1-along midline (B); 2-between consecutive plates or rings (respectively, B and C), as already suggested but not shown by Babcock et al.[5]; and 3-between layers (‘peeling’) (D and E; arrowheads in E indicate the two layers of the wall), also occurring within a single sclerite (D, centre of the image). D: CL image of tube longitudinal section of

sample in C (the larger one; dashed line indicates cutting direction that yielded section in D) depicting a single layer with partial preservation of the bilayered microstructure (centre of the image). A single large ring is shown at the right (arrowhead). F: CL image in D with indication of original image assembling. Arrowhead points to a thick element. Scale bars: A and B = 4 mm; C = 2 mm; D-F = 0.2 mm. Original figures have been cropped to remove excess of empty space around the fossils and also to enable stitching (D and F).

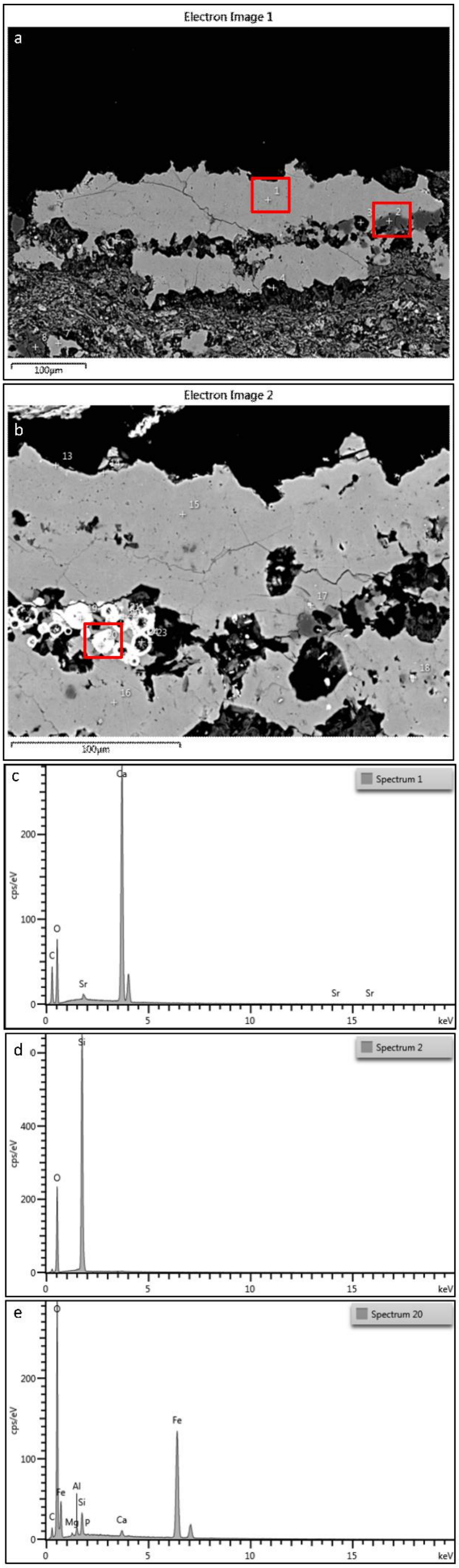

Electron Image 1
a
100µm
Electron Image 2
b
100µm
c
Spectrum 1
cps/eV
keV
d
Spectrum 2
cps/eV
keV
e
Spectrum 20
cps/eV
keV

Figure S9. BSE and EDS of *Corumbella* skeleton, related to Fig. 3. A and B: skeletal rings of Sample CAP/1A 1022 (Fig. 3C; Figs. S3B; S4). Position of EDS point spectra in C-E is highlighted in A and B by red rectangles. EDS point spectra of non-silicified and silicified skeletal regions (C and D, respectively), and of pyrite pseudoframboid (E).

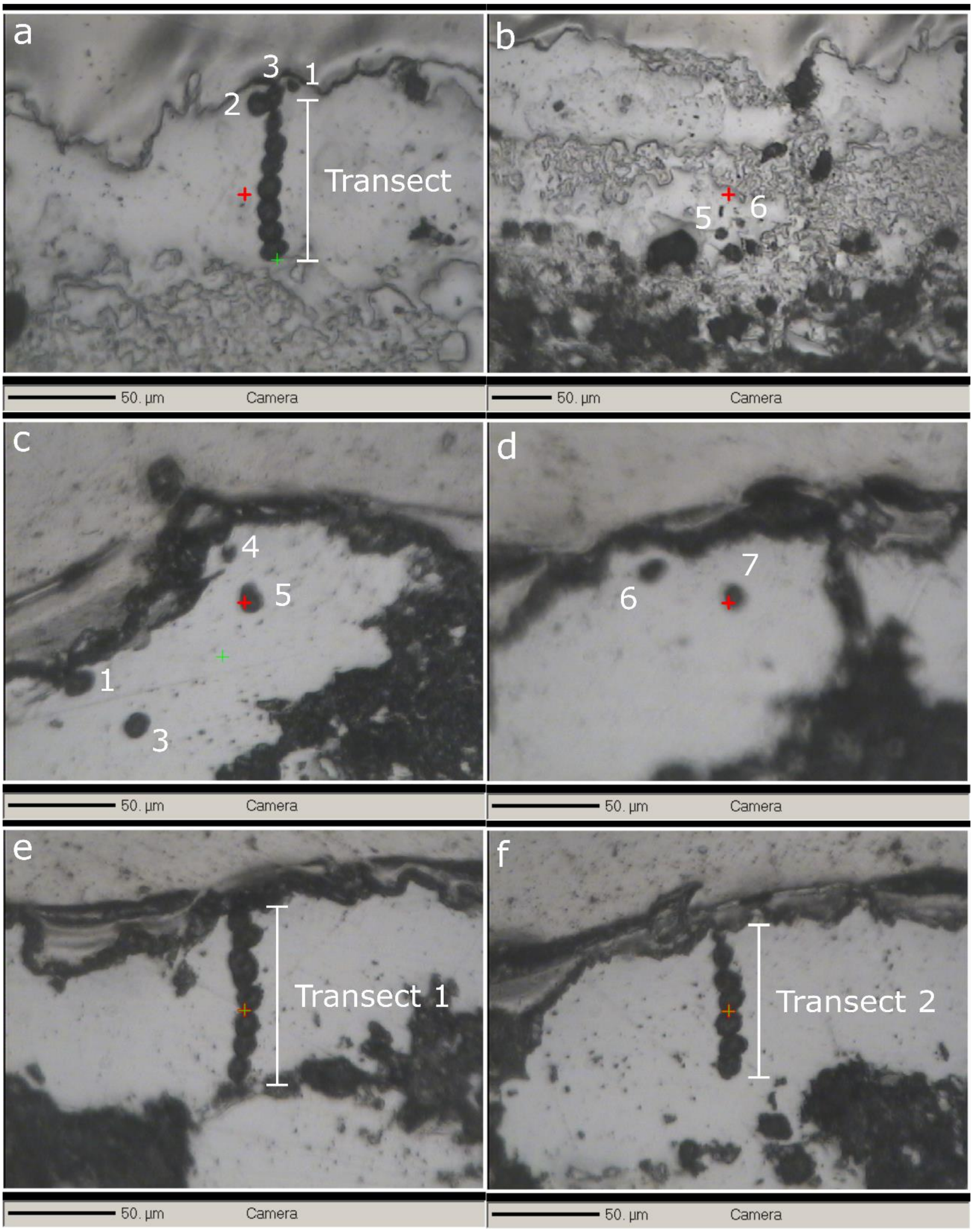

Figure S10. Points analysed with EPMA, related to Figs. 1, 3 and 4. A and B: CAP/1A 1022. C-F: CAP/1F 11. Measurements are available in Table S6.

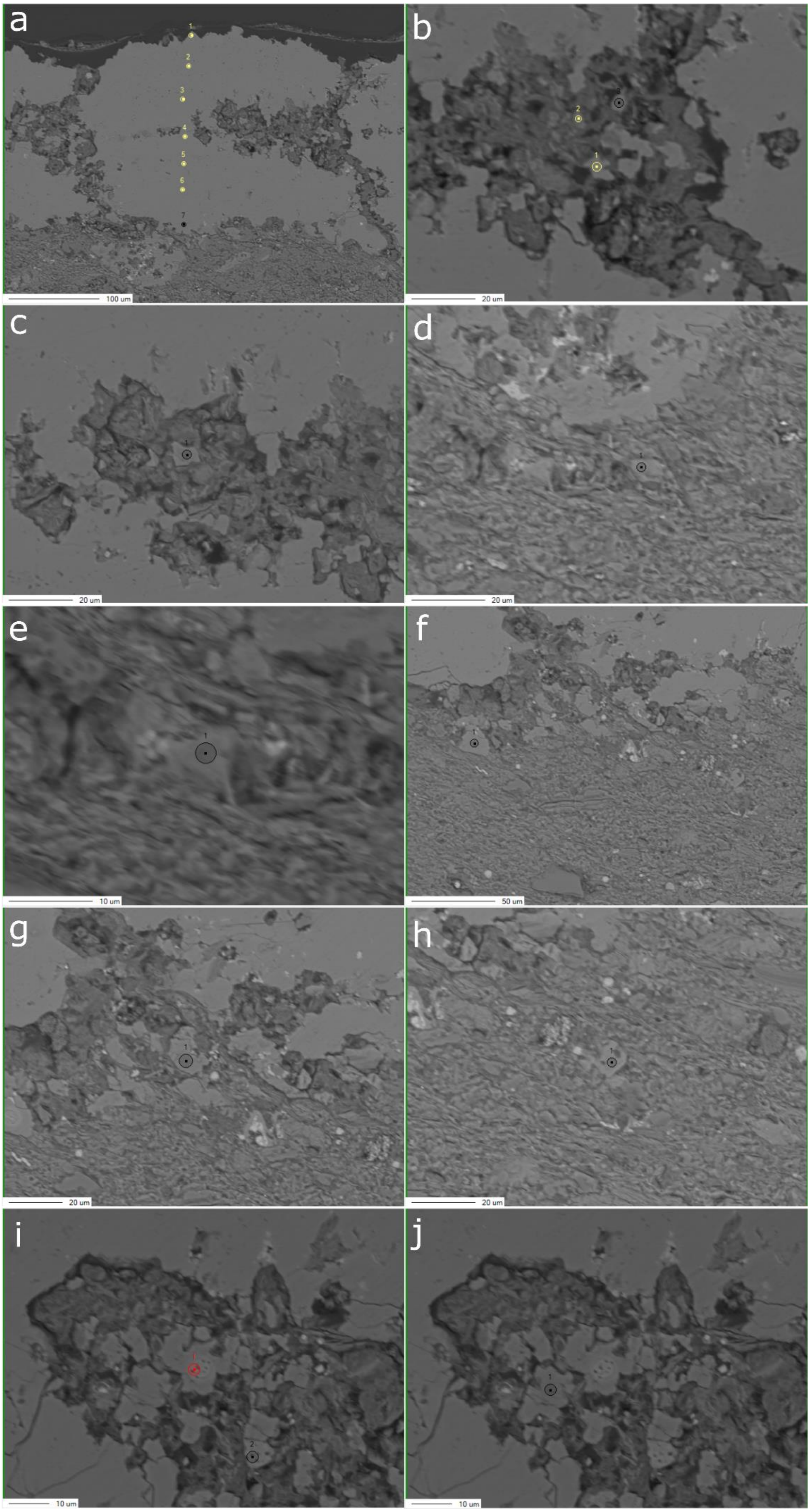
a
100 um
b
20 um
c
20 um
d
20 um
e
10 um
f
50 um
g
20 um
h
20 um
i
10 um
j
10 um

Figure S11. Points analysed with EPMA, related to Fig. 1. A-I: CAP/1F 11. Measurements are available in Table S7. Original figures have been cropped to remove excess of empty space.

Table S3. Comparison of *Corumbell*a and Ediacaran skeletonized biota features, related to Figs. 1-4.

Features			*Corumbella*	Ediacaran skeletonized metazoans			
				Sinotubulitids (*Sinotubulites*)	Cloudinids	*Gaojiashania*	*Namacalathus*
Body organization	Growth polarity	Antero-posterior axis	X				
		Regular imbrication mode of the sclerites in each layer	X				
	Variation of morphology along the tube (uniseriate proximal region grades to quadriseriate distal region)		X				
	Proximal region with circular cross-section and more distal region with polyhedral cross-section		X	Polygonal cross-section [32]	Circular cross-section	Circular cross-section	
Organization of building elements	Multi-element articulated skeleton (plates/rings are non-continuous building unities, either isolated or, more commonly, imbricated, i.e. cataphract)		X	Continuous irregular annulations in a multilayered 'tube-in-tube' construction and annulations restricted to the surface of the tube (Fig. 3B of Chen et al.[32])	Skeleton with 'funnel-in-funnel' organization and collars[33,34]. Continuous, internally smooth and single-layered wall. *Costatubus* does not have collars and is a cylinder formed by slightly imbricated barrel-shaped elements, like the proximal region of *Corumbella*, but *Costatubus* has internally smooth wall and still undetermined biomineralization[35]	Tube built by the alternation of rings and buckets with taphonomically imbricated rings[39]	Continuous wall[40]
	Sclerites, with sigmoidal cross-section		X				
	Alternation of plates along the midlines		X				
	Serially repeated plates/rings		X				
	Faces with midlines and lateral edges		X				
	Midlines are continuous articulation axes of discontinuous converging plates		X				
	Wall with two layers		X				
	Inner tube wall is irregular, i.e. not smooth (due to sclerite imbrication)		X				
	Internal thickening structures						
Microfabric and composition	Intraskeletal lamination of mineral-rich and organic-rich laminae			Possibly composite skeleton of aragonite and organics (Chen et al.[32])	*Cloudina*: Lamination of organic-rich and mineral-rich laminae (could not be original), with purportedly aragonitic[36,37](; but see Yang et al.[34]) or fibrous high-Mg calcite original preservation (currently preserved by granular calcitic microfabric)[38]	Controversial composition[39]	Regularly foliated microfabric with tripartite structuring and cone-in-cone deflections: two laminar layers sandwiching a middle layer of rod-like microdolomite[40]. Either undetermined[41], high-Mg calcite[40] or aragonitic[36] original mineralogy
	Possibly originally laminar microfabric formed by aragonite crystals embedded in an organic matrix		X				
	Preferential crystallographic orientation: C-axis of calcite crystals are co-aligned with the <10-14> axes, both oriented perpendicular to the tube length		X				
	Composite skeletal composition (i.e. crystals embedded in organics): Organic and aragonitic		X				
	Biologically controlled biomineralization		X				
Symmetry	Four-fold symmetry		X				Radial
Ecology	Sessile		X	Epifaunal? (Chen et al., 2008)	X	X	X

Table S4. Comparison of *Corumbell*a and polychaete (annelids) features, related to Figs. 1-4.

Features			*Corumbella*	Lophotrochozoa			
				Annelida			
				Siboglinids	Sabellids	Cirratulitids	Serpulids
Body organization	Growth polarity	Antero-posterior axis	X	X	X	X	X
		Regular imbrication mode of the sclerites in each layer	X				
	Variation of morphology along the tube (uniseriate proximal region grades to quadriseriate distal region)		X				
	Proximal region with circular cross-section and more distal region with polyhedral cross-section		X			Polygonal cross-section	Polygonal cross-section
Organization of building elements	Multi-element articulated skeleton (plates/rings are non-continuous building unities, either isolated or, more commonly, imbricated, i.e. cataphract)		X	Tube with continuous wall	Continuous tube wall with continuous wrinkles in tube longitudinal section[44]	Tube with continuous lamellar wall with tabulae[45]	Tube with continuous wall
	Sclerites, with sigmoidal cross-section		X				
	Alternation of plates along the midlines		X				
	Serially repeated plates/rings		X				
	Faces with midlines and lateral edges		X				
	Midlines are continuous articulation axes of discontinuous converging plates		X				
	Wall with two layers		X				
	Inner tube wall is irregular, i.e. not smooth (due to sclerite imbrication)		X				
	Internal thickening structures						
Microfabric and composition	Intraskeletal lamination of mineral-rich and organic-rich laminae			Unmineralized tubes that are secondary mineralized, with mineral growth either by the interaction with microorganisms[42], or post-mortem[43]	Double-layered wall formed by an outer spherulitic layer and an internal layer of spherulitic prisms, both layers being separated by organic layers[44]. Composite skeleton of aragonite	Microstructure is similar to that of the inner layer of sabeliids; aragonitic composition[44]	'Chevron' arrangement of crystal fabrics, as well as irregularly oriented prisms[44]. Organic-rich and mineral-rich laminae, composite, aragonite (also calcite), biologically controlled biomineralization[46]
	Possibly originally laminar microfabric formed by aragonite crystals embedded in an organic matrix		X				
	Preferential crystallographic orientation: C-axis of calcite crystals are co-aligned with the <10-14> axes, both oriented perpendicular to the tube length		X				
	Composite skeletal composition (i.e. crystals embedded in organics): Organic and aragonitic		X				
	Biologically controlled biomineralization		X				
Symmetry	Four-fold symmetry		X	Bilateral	Bilateral	Bilateral	Bilateral
Ecology	Sessile		X	X	X	X	X

Table S5. Data for identification of Raman spectra, related to Fig. 3. References: 1- de Faria, Silva and Oliveira[47] (1997); 2- Urmos, Sharma and Mackenzie[48]; 3- Gunasekaran, Anbalagan and Pandi[49]; 4- Jehlicka, Urban and Pokorný[50]; 5- Ferralis et al.[51].

Spectral Range (cm^{-1})	Vibrational Mode	Spectra Interpretation	References
300-700	E_g(Fe-O)	Fe-oxyhydr(oxides)	1
1080-1090	ν_1(C-O)	Carbonate	2, 3
1500-1600	ν_1(C-C)	G band of amorphous C	4, 5

Table S6. Data from electron probe micro analysis (EPMA) experiments, related to Figs. 1, 3 and 4. Negative values are below the detection limit. Data in ppm.

Sample	Measurement	Ca	Mg	Mn	Fe	P	Sr	O
CAP/1A 1022	1	370,219	929	3005	2159	57	6354	151,131
	2	389,359	176	5	137	62	10,701	157,623
	3	378,965	577	2691	1524	34	8175	154,420
	Transect	389,953	229	-0.0135	1116	81	8598	157,775
		379,584	135	-0.0082	106	69	10,737	153,675
		388,182	182	-0.0099	106	42	11,567	157,250
		380,694	115	-0.0075	256	72	14,001	154,749
		390,075	259	32	98	75	11,977	158,209
		389,659	151	-0.0058	227	68	10,009	157,614
		387,008	125	0.0000	288	30	12,678	157,013
		388,285	228	5	977	85	10,784	157,513
	5	383,069	476	87	1419	46	13,087	156,116
	6	376,869	706	844	1678	36	13,180	154,092
CAP/1F 11	1	392,272	256	81	1195	39	9923	158,991
	3	395,325	154	2	147	20	13,260	160,405
	4	390,994	143	8	272	64	11,778	158,492
	5	388,883	246	138	114	15	16,996	158,600
	6	377,062	1031	3506	2214	26	6,260	154,035
	7	387,083	208	132	162	63	11,224	156,876
	Transect 1	391,265	790	1424	865	92	10,455	159,404
		390,922	177	79	189	40	13,404	158,749
		392,259	243	-0.0056	91	46	14,076	159,389
		393,432	247	27	38	57	10,508	159,232
		392,523	343	117	95	21	11,947	159,192
		396,638	231	69	151	45	11,749	160,758
		394,039	189	-0.0014	148	34	10,253	159,379
		393,143	339	235	340	55	12,226	159,634
	Transect 2	310,756	3496	2726	3695	66	6,119	129,410
		390,932	221	197	362	27	10,634	158,343
		391,784	195	-0.0039	60	59	10,818	158,586
		389,544	205	166	114	39	11,413	157,856
		392,659	217	62	63	109	10,757	159,034
		392,719	283	129	34	58	10,855	159,064
		387,532	153	-0.0046	17	19	12,818	157,161

Table S7. Data from electron probe micro analysis (EPMA) experiments, related to Fig. 1. Data in ppm.

Sample	Measurement	Figure	Region	Si	Al	Fe	Mn	P	Ca	Sr	Mg	O
CAP/1F 11	1	S12 A	Fossil-Transect	211	285	2221	3613	0	386400	6579	921	158300
	2			0	4	167	0	108	383900	9514	138	155500
	3			0	0	123	47	280	375900	13300	46	152900
	4			0	2	712	57	24	388300	12700	111	157700
	5			0	0	227	0	26	365900	12100	13	148600
	6			0	0	110	16	0	381400	11200	37	154300
	7			571	67	1081	126	356	383000	9019	178	156200
	1	S12 B	Host rock-Carbonate	14500	7115	2861	1098	0	329300	4792	2850	158200
	2	S12 B		217900	114900	10600	0	153	5614	0	19000	368500
	3	S12 B		26400	13800	3622	760	39	288800	3583	4200	162400
	1	S12 C		11000	5947	1221	271	336	353800	845	2064	161500
	1	S12 D		28300	3634	5737	2389	140	318000	4732	2336	167300
	1	S12 E		17700	9978	5105	3058	173	334100	5962	2564	167800
	1	S12 F		80	213	1485	64	91	384300	6904	804	156100
	1	S12 G		7441	1110	2060	0	134	352200	0	1654	151900
	1	S12 H		55300	20600	5590	2270	14	268800	3717	6196	195600
	1	S12 I		194	215	619	0	407	365600	149	1208	147900
	2	S12 I		4538	2818	1094	96	0	361000	189	1435	153100
	1	S12 J		1717	1215	742	0	54	363300	153	1094	149100

Table S8. Detection limits of data from Table S6, related to Figs. 1, 3 and 4. Detection limits by weight in ppm.

Sample	Measurement	Ca	Mg	Mn	Fe	P	Sr
CAP/1A 1022	1	2171.	28.	207.	137.	52.	146.
	2	2181.	29.	181.	131.	54.	149.
	3	2045.	29.	206.	138.	55.	150.
	Transect	2165.	30.	188.	135.	55.	154.
		2189.	31.	188.	138.	57.	158.
		2124.	30.	184.	132.	57.	156.
		2044.	30.	186.	132.	56.	155.
		2038.	30.	182.	128.	57.	149.
		2078.	30.	181.	127.	57.	153.
		2092.	30.	181.	129.	55.	155.
		2075.	30.	182.	130.	54.	149.
	5	2166.	29.	183.	139.	55.	157.
	6	2132.	30.	184.	140.	56.	159.
CAP/1F 11	1	2126.	30.	182.	130.	56.	152.
	3	2047.	30.	183.	129.	58.	150.
	4	2102.	29.	181.	136.	54.	159.
	5	2125.	30.	175.	132.	56.	158.
	6	2109.	30.	215.	128.	54.	141.
	7	2162.	30.	178.	132.	57.	158.
	Transect 1	2023.	30.	193.	135.	55.	151.
		2170.	30.	178.	131.	58.	156.
		2224.	30.	189.	135.	57.	160.
		2120.	29.	177.	130.	55.	151.
		2186.	30.	175.	130.	55.	153.
		2106.	30.	179.	129.	58.	153.
		2120.	30.	181.	131.	56.	158.
		2105.	30.	178.	134.	56.	154.

	Transect 2	1946.	30.	207.	138.	53.	155.
		2147.	29.	180.	133.	55.	155.
		2053.	30.	181.	133.	57.	156.
		2086.	30.	177.	132.	58.	156.
		2139.	30.	177.	129.	57.	155.
		2083.	30.	174.	130.	55.	153.
		2169.	30.	185.	131.	58.	154.

Table S9. Detection limits of data from Table S7, related to Fig. 1. Detection limits in ppm.

Sample	Measurement	Figure	Region	Si	Al	Fe	Mn	P	Ca	Sr	Mg
CAP/1F 11	1	S12 A	Fossil-Transect	192	93	116	94	269	176	208	90
	2			182	91	110	99	251	172	217	88
	3			196	89	114	94	190	182	200	92
	4			191	91	112	99	263	172	221	92
	5			186	89	112	97	255	174	218	91
	6			183	92	113	96	282	176	194	88
	7			191	92	117	98	191	177	222	92
	1	S12 B	Host rock-Carbonate	183	101	103	75	262	289	166	73
	2	S12 B		160	127	91	73	209	178	305	75
	3	S12 B		167	99	98	67	245	269	186	76
	1	S12 C		183	98	100	75	186	291	162	72
	1	S12 D		193	110	99	73	224	269	195	67
	1	S12 E		188	103	106	78	242	299	179	75
	1	S12 F		189	102	101	76	260	280	168	69
	1	S12 G		182	98	103	76	229	279	183	70
	1	S12 H		171	102	105	74	241	284	197	71
	1	S12 I		173	96	103	75	186	291	154	70
	2	S12 I		172	97	98	74	265	277	165	72
	1	S12 J		179	100	104	76	229	315	151	73

Table S10. EPMA processed data from samples CAP/1A 1022 and CAP/1F 11, related to Figs. 1, 3 and 4, as well as to STAR Methods.

Element	Region analysed (ppm)											
	Margin-Sclerite				Inner region-Sclerite				Host rock-Carbonate			
	Min	Max	Mean	Standard Deviation	Min	Max	Mean	Standard Deviation	Min	Max	Mean	Standard Deviation
Si	211	571	391	180	0	0	0	0	80	217900	32090	58025
Al	67	285	176	109	0	4	1	1	213	114900	15128	30659
Ca	310760	392270	340490	29730	365900	396638	388200	6344	5614	384300	310400	97282
Mg	143	3496	160	17	13	343	185	77	804	19000	3783	4810
Mn	0	3613	44	36	0	235	75	69	0	3058	833	1069
Fe	272	3695	568	296	17	977	195	198	619	10600	3394	2823
P	0	356	13	13	0	280	56	49	0	407	128	123
Sr	6119	13180	6190	70	9514	16996	11870	1529	0	6904	2585	2513
O	129410	159404	140270	10860	148600	160758	157410	2519	147900	368500	178280	58628

Supplemental references list

1. Pacheco, M. L. A. F., Galante, D., Rodrigues, F., Leme, J. M., Bidola, P., Hagadorn, W., Stockmar, M., Herzen, J., Rudnitzki, I. D., Pfeiffer, F., and Marques, A. C. (2015). Insights into the skeletonization, lifestyle, and affinity of the unusual Ediacaran fossil *Corumbella*. PLoS ONE. DOI: 10.1371/journal. pone.0114219.

2. Parry, L. A., Boggiani, P. C., Condon, D. J., Garwood, R. J., Leme, J. M., McIlroy, D., Brasier, M. D., Trindade, R., Campanha, G. A. C., Pacheco, M. L. A. F., Diniz, C. Q. C., and Liu, A. G. (2017). Ichnological evidence for meiofaunal bilaterians from the terminal Ediacaran and earliest Cambrian of Brazil. Nat. Ecol. Evol. **1**. DOI: 10.1038/s41559-017-0301-9.

3. Oliveira R. S. (2010). Depósitos de Rampa Carbonática Neoproterozóica do Grupo Corumbá, Região de Corumbá, Mato Grosso, masters dissertation, Instituto de Geociências, Universidade Federal do Pará.

4. Morais L. P. C. (2013). Paleobiologia da Formação Bocaina (Grupo Corumbá), Ediacarano, Mato Grosso do Sul, masters dissertation, Instituto de Geociências, Universidade de São Paulo, São Paulo.

5. Babcock, L. E., Grunow, A. M., Sadowski, G. R., and Leslie, S. A. (2005). *Corumbella*, an Ediacaran-grade organism from the Late Neoproterozoic of Brazil. Palaeogeogr. Palaeoclimatol. Palaeoecol. **220 (1-2),** 7–18. DOI: 10.1016/j.palaeo.2003.01.001.

6. Warren, L. V., Pacheco, M. L. A. F., Fairchild, T. R., Simões, M. G., Riccomini, C., Boggiani, P. C., and Cáceres, A. A. (2012). The dawn of animal skeletogenesis: ultrastructural analysis of the Ediacaran metazoan *Corumbella werneri*. Geology **40 (8),** 691–694. DOI: 10.1130/G33005.1.

7. Mendoza-Becerril, M. A., Maronna, M. M., Pacheco, M. L. A. F., Simões, M. G., Leme, J. M., Miranda, L. S., Morandini, A. C., and Marques, A. C. (2016). An evolutionary comparative analysis of the medusozoan (Cnidaria) exoskeleton. Zool. J. Linnean Soc. **178 (2),** 206-225. DOI: 10.1111/zoj.12415.

8. Van Iten, H., Leme, J. M., Pacheco, M. L. A. F., Simões, M. G., Fairchild, T.R., Rodrigues, F., Galante, D., Boggiani, P. C., and Marques, A. C. (2016). Origin and early diversification of phylum Cnidaria: key macrofossils from the Ediacaran system of North and South America. In The Cnidaria, Past, Present and Future: The World of Medusa and Her sisters, S. Goffredo and Z. Dubinsky, eds. (Springer), pp. 31-40.

9. Walde, D. H.-G., Weber, B., Erdtmann, B.-D., and Steiner, M. (2019). Taphonomy of *Corumbella werneri* from the Ediacaran of Brazil: sinotubulitid tube or conulariid test? Alcheringa **43 (3),** 335–350. DOI: 10.1080/03115518.2019.1615551.

10. Van Iten, H., Marques, A. C., Leme, J. M., Pacheco, M. L. A. F., and Simões, M. G. (2014). Origin and early diversification of the phylum Cnidaria Verrill: major developments in the analysis of the taxon's Proterozoic–Cambrian history. Palaeontology **57 (4),** 677-690. DOI: 10.1111/pala.12116.

11. Ford, R. C., Van Iten, H., and Clark, G. R. II (2016). Microstructure and composition of the periderm of conulariids. J. Paleontol. **90 (3),** 389-399. DOI: 10.1017/jpa.2016.63.

12. Sendino, C., Zágoršek, K., and Taylor, P. D. (2012). Asymmetry in an Ordovician conulariid cnidarian. Lethaia **45 (3),** 423–431. DOI: 10.1111/j.1502-3931.2011.00302.x.

13. Lowenstam, H. A., and Weiner, S. (1989). On Biomineralization (Oxford University Press).

14. Cuif, J.-P., Dauphin, Y., and Sorauf, J. E. (2011). Biominerals and Fossils Through Time (Cambridge University Press).

15. Cairns, S. D. (2011). Global diversity of the Stylasteridae (Cnidaria: Hydrozoa: Athecatae). PLoS ONE **6 (7): e21670,** 1-13. DOI: 10.1371/journal.pone.0021670.

16. Sorauf, J. E. (1980). Biomineralization, structure and diagenesis of the coelenterate skeleton. Acta Palaeontol. Pol. **25 (3-4),** 327-343.

17. Miyazaki, Y., and Reimer, J. D. (2015). A new genus and species of octocoral with aragonite calcium-carbonate skeleton (Octocorallia, Helioporacea) from Okinawa, Japan. ZooKeys **511,** 1-23. DOI: 10.3897/zookeys.511.9432.

18. Perrin, J., Vielzeuf, D., Ricolleau, A., Dallaporta, H., Valton, S., and Floquet, N. (2015). Block-by-block and layer-by-layer growth modes in coral skeletons. Am. Mineral. **100 (4),** 681-695. DOI: 10.2138/am-2015-4990.

19. Guzman, C., Shinzato, C., Lu, T.-M., and Conaco, C. (2018). Transcriptome analysis of the reef-building octocoral, *Heliopora coerulea*. Sci. Rep. **8, 8397**. DOI:10.1038/s41598-018-26718-5.

20. Tuti, Y., and van Ofwegen, L. P. (2018). Gorgonians in Indonesian Waters (PT. Media Sains Nasional).

21. Song, J.-I., Hwang, S.-J., Moon, H., and An, I.-Y. (2012). Taxonomic study of suborder Calcaxonia (Alcyonacea: Octocorallia: Anthozoa) from King Sejong Station, Antarctic. Anim. Syst. Evol. Divers. **28 (2),** 84-96. DOI: 10.5635/ASED.2012.28.2.084.

22. Cairns, S. D. (2018). Deep-water octocorals (Cnidaria, Anthozoa) from the Galápagos and Cocos Islands. Part 1: Suborder Calcaxonia. ZooKeys **729,** 1-46. DOI: 10.3897/zookeys.729.21779.

23. Stolarski, J., Bosellini, F. R., Wallace, C. C., Gothmann, A. M., Mazur, M., Domart-Coulon, I., Gutner-Hoch, E., Neuser, R. D., Levy, O., Shemesh, A., and Meibom, A. (2016). A unique coral biomineralization pattern has resisted 40 million years of major ocean chemistry change. Sci. Rep. **6, 27579**. DOI: 10.1038/srep27579.

24. Stampar, S. N., Beneti, J. S., Acuña, F. H., and Morandini, A. C. (2015). Ultrastructure and tube formation in Ceriantharia (Cnidaria, Anthozoa). Zool. Anz. **254 (1),** 67-71. DOI: 10.1016/j.jcz.2014.11.004.

25. Von Euw, S., Zhang, Q., Manichev, V., Murali, N., Gross, J., Feldman, L. C., Gustafsson, T., Flach, C., Mendelsohn, R., and Falkowski, P. G. (2017). Biological control of aragonite formation in stony corals. Science **356 (6341),** 933–938. DOI: 10.1126/science.aam6371.

26. Kingsley, R. J., and Watabe, N. (1982). Ultrastructural investigation of spicule formation in the gorgonian *Leptogorgia virgulata* (Lamarck) (Coelenterata: Gorgonacea). Cell Tissue Res. **223 (2),** 325-334. DOI: 10.1007/BF01258493.

27. Coronado, I., Pérez-Huerta, A., and Rodríguez, S. (2015). Crystallographic orientations of structural elements in skeletons of Syringoporicae (Tabulate corals, Carboniferous): implications for biomineralization processes in Palaeozoic corals. Palaeontology **58 (1),** 111-132. DOI: 10.1111/pala.12127.

28. Sethmann, I., Helbig, U., and Wörheide, G. (2007). Octocoral sclerite ultrastructures and experimental approach to underlying biomineralisation principles. Cryst. Eng. Comm. **9,** 1262-1268. DOI: 10.1039/B711068E.

29. Cuif, J.-P., Dauphin, Y., Nehrke, G., Nouet, J., and Perez-Huerta, A. (2012). Layered growth and crystallization in calcareous biominerals: impact of structural and chemical evidence on two major concepts in invertebrate biomineralization studies. Minerals **2 (1),** 11-39. DOI: 10.3390/min2010011.

30. Stampar, S. N., Maronna, M. M., Kitahara, M. V., Reimer, J. D., Beneti, J. S., and Morandini, A. C. (2016). Ceriantharia in current systematics: life cycles, morphology and genetics. In The

Cnidaria, Past, Present and Future: The World of Medusa and Her Sisters, S. Goffredo and Z. Dubinsky, eds. (Springer), pp. 61-72.

31. McFadden, C. S., Quattrini, A. M., Brugler, M. R., Cowman, P. F., Dueñas, L. F., Kitahara, M. V., Paz-García, D. A., Reimer, J. D., and Rodríguez, E. (2021). Phylogenomics, origin, and diversification of anthozoans (Phylum Cnidaria). Syst. Biol. **70 (4),** 635-647. DOI: 10.1093/sysbio/syaa103.

32. Chen, Z., Bengtson, S., Zhou, C. M., Hua, H., and Yue, Z. (2008). Tube structure and original composition of *Sinotubulites*: shelly fossils from the late Neoproterozoic in southern Shaanxi, China. Lethaia **41 (1),** 37–45. DOI: 10.1111/j.1502-3931.2007.00040.x.

33. Hua, H., Chen, Z., Yuan, X., Zhang, L., and Xiao, S. (2005). Skeletogenesis and asexual reproduction in the earliest biomineralizing animal *Cloudina*. Geology **33 (4),** 277-280. DOI: 10.1130/G21198.1.

34. Yang, B., Steiner, M., Schiffbauer, J. D., Selly, T., Wu, X., Zhang, C., and Liu, P. (2020). Ultrastructure of Ediacaran cloudinids suggests diverse taphonomic histories and affinities with non-biomineralized annelids. Sci. Rep. **10**. DOI: https://doi.org/10.1038/s41598-019-56317-x.

35. Selly, T., Schiffbauer, J. D., Jacquet, S. M., Smith, E. F., Nelson, L. L., Andreasen, B. D., Huntley, J. W., Strange, M. A., O'Neil, G. R., Thater, C. A. et al. (2020). A new cloudinid fossil assemblage from the terminal Ediacaran of Nevada, USA. J. Syst. Palaeontol. **18 (4),** 357-379. DOI: 10.1080/14772019.2019.1623333.

36. Pruss, S. B., Blättler, C. L., Macdonald, F. A., and Higgins, J. A. (2018). Calcium isotope evidence that the earliest metazoan biomineralizers formed aragonite shells. Geology **46 (9),** 763–766. DOI: 10.1130/G45275.1.

37. Gilbert, P. U. P. A., Porter, S. M., Sun, C.-Y., Xiao, S., Gibson, B. M., Shenkar, N., and Knoll, A. H. (2019). Biomineralization by particle attachment in early animals. Proc. Natl. Acad. Sci. U.S.A. **116 (36),** 17659-17665. DOI:10.1073/pnas.1902273116.

38. Zhuravlev, A. Yu., Liñán, E., Gámez Vintaned, J.A., Debrenne, F., and Fedorov, A. B. (2012). New finds of skeletal fossils in the terminal Neoproterozoic of the Siberian Platform and Spain. Acta Palaeontol. Pol. **57 (1),** 205–224. DOI: 10.4202/app.2010.0074.

39. Cai, Y., Hua, H., and Zhang, X. (2013). Tube construction and life mode of the Late Ediacaran tubular fossil *Gaojiashania cyclus*. Prec. Res. **224,** 255-267. DOI: 10.1016/j.precamres.2012.09.022.

40. Zhuravlev, A. Yu., Wood, R. A., and Penny, A. M. (2015). Ediacaran skeletal metazoan interpreted as a lophophorate. Proc. R. Soc. B. **282 (1818)**. DOI: http://dx.doi.org/10.1098/rspb.2015.1860.

41. Porter, S. M. (2010). Calcite and aragonite seas and the de novo acquisition of carbonate skeletons. Geobiology **8 (4),** 256–277. DOI: 10.1111/j.1472-4669.2010.00246.x.

42. Rincón-Tomás, B., Somoza, L., Sauter, K., Hause-Reitner, D., Madureira, P., Schneider, D., González, F. J., Medialdea, T., Carlsson, J., Reitner, J., and Hoppert, M. (2019). New insights into Siboglinidae microbiota – external tube contributes to an increment of the total microbial biomass. Preprint at https://peerj.com/preprints/27730/.

43. Haas, A., Little, C. T. S., Sahling, H., Bohrmann, G., Himmler, T., and Peckmann, J. (2009). Mineralization of vestimentiferan tubes at methane seeps on the Congo deep-sea fan. Deep-Sea Res. Part I: Oceanogr. Res. Pap. **56 (2),** 283–293. DOI: 10.1016/j.dsr.2008.08.007.

44. Vinn, A., Ten Hove, H. A., and Mutvei, H. (2008). On the tube ultrastructure and origin of calcification in Sabellids (Annelida, Polychaeta). Palaeontology **51 (2),** 295–301. DOI: 10.1111/j.1475-4983.2008.00763.x.

45. Vinn, A., Ten Hove, H. A., Mutvei, H., and Kirsimäe, K. (2008). Ultrastructure and mineral composition of serpulid tubes (Polychaeta, Annelida). Zool. J. Linnean Soc. **154 (4),** 633–650. DOI: 10.1111/j.1096-3642.2008.00421.x.

46. Fischer, R., Pernet, B., and Reitner, J. (2000). Organomineralization of Cirratulid Annelid tubes - Fossil and Recent examples. Facies **42,** 35-50. DOI: 10.1007/BF02562565.

47. de Faria, D. L. A., Silva, S. V., and Oliveira, M. T. (1997). Raman microspectroscopy of some iron oxides and oxyhydroxides. J. Raman Spectrosc. **28 (11),** 873-879. DOI: 10.1002/(SICI)1097-4555(199711)28:11<873::AID-JRS177>3.0.CO;2-B.

48. Urmos, J., Sharma, S. K., and Mackenzie, F. T. (1991). Characterization of some biogenic carbonates with Raman spectroscopy. Am. Mineral. **76 (3-4),** 641-646.

49. Gunasekaran, S., Anbalagan, G., and Pandi, S. (2006). Raman and infrared spectra of carbonates of calcite structure. J. Raman Spectrosc. **37 (9),** 892-899. DOI: 10.1002/jrs.1518.

50. Jehlicka, J., Urban, O., and Pokorný, J. (2003). Raman spectroscopy of carbon and solid bitumens in sedimentary and metamorphic rocks. Spectrochim. Acta A **59 (10),** 2341-2352.

51. Ferralis, N., Matys, E. D., Knoll, A. H., Hallman, C., and Summons, R. E. (2016). Rapid, direct and non-destructive assessment of fossil organic matter via microRaman spectroscopy. Carbon **108,** 440-449. DOI: 10.1016/j.carbon.2016.07.039.

52. Vinther, J., Van Roy, P., and Briggs, D. E. G. (2008). Machaeridians are Palaeozoic armoured annelids. Nature **451,** 185-188. DOI: 10.1038/nature06474.

53. Yang, X., Vinn, O., Hou, X., and Tian, X. (2013). New tubicolous problematic fossil with some “lophophorate” affinities from the Early Cambrian Chengjiang biota in south China. GFF **135 (2),** 184-190. DOI: 10.1080/11035897.2013.801035.

54. Zhang, Z., Holmer, L. E., Skovsted, C. B., Brock, G. A., Budd, G. E., Fu, D., Zhang, X., Shu, D., Han, J., and Liu, J. (2013). A sclerite-bearing stem group entoproct from the early Cambrian and its implications. Sci. Rep. **3, 1066**. DOI:10.1038/srep01066.

55. Skovsted, C. B., Brock, G. A., Paterson, J. R., Holmer, L. E., and Budd, G. E. (2008). The scleritome of *Eccentrotheca* from the Lower Cambrian of South Australia: Lophophorate affinities and implications for tommotiid phylogeny. Geology **36 (2),** 171-174. DOI: 10.1130/G24385A.1.

56. Skovsted, C. B., Brock, G. A., Topper, T. P., Paterson, J. R., and Holmer, L. E. (2011). Scleritome construction, biofacies, biostratigraphy and systematics of the Tommotiid *Eccentrotheca helenia* sp. nov. from the Early Cambrian of south Australia. Palaeontology **54 (2),** 253-286. DOI: 10.1111/j.1475-4983.2010.01031.x.

57. Skovsted, C. B., Holmer, L. E., Larsson, C. M., Högström, A. E. S., Brock, G. A., Topper, T. P., Balthasar, U., Stolk, S. P., and Paterson, J. R. (2009). The scleritome of *Paterimitra*: an Early Cambrian stem group brachiopod from South Australia. Proc. R. Soc. B **276 (1662),** 1651–1656. DOI: 10.1098/rspb.2008.1655.

58. Skovsted, C. B., Balthasar, U., Brock, G. A., and Paterson, J. R. (2009). The tommotiid *Camenella reticulosa* from the Early Cambrian of South Australia: morphology, scleritome reconstruction, and phylogeny. Acta Palaeontol. Pol. **54 (3),** 525-540. DOI: 10.4202/app.2008.0082.

59. Morris, S. C. (2001) Significance of early shells. In Palaeobiology II, D. E. G. Briggs, and P. R. Crowther, eds. (Blackwell Publishing Ltd.), pp. 31-40.

60. Balthasar, U., Cusack, M., Faryma, L., Chung, P., Holmer, L. E., Jin, J., Percival, I. G., and Popov, L. E. (2011). Relic aragonite from Ordovician–Silurian brachiopods: implications for the evolution of calcification. Geology **39 (10),** 967–970. DOI: 10.1130/G32269.1.

61. Checa, A. G. (2018). Physical and biological determinants of the fabrication of molluscan shell microstructures. Front. Mar. Sci. **5:353,** DOI: 10.3389/fmars.2018.00353.

62. Taylor, P. D., Kudryavtsev, A. B., and Schopf, J. W. (2008). Calcite and aragonite distributions in the skeletons of bimineralic bryozoans as revealed by Raman spectroscopy. Invertebr. Biol. **127 (1),** 87-97. DOI: 10.1111/j.1744-7410.2007.00106.x.

63. Taylor, P. D., Lombardi, C., and Cocito, S. (2014). Biomineralization in bryozoans: present, past and future. Biol. Rev. **90 (4),** 1118–1150. DOI: 10.1111/brv.12148.

64. Taylor, P. D., and Weedon, M. J. (2000). Skeletal ultrastructure and phylogeny of cyclostome bryozoans. Zool. J. Linnean Soc. **128 (4),** 337–399. DOI: 10.1006/zjls.1999.0195.

65. Vendrasco, M. J., Porter, S. M., Kouchinsky, A., Li, G., and Fernandez, C. Z. (2010). New data on molluscs and their shell microstructures from the middle Cambrian Gowers Formation, Australia. Palaeontology **53 (1),** 97-135. DOI: 10.1111/j.1475-4983.2009.00922.x.

66. Vendrasco, M. J., Rodríguez-Navarro, A. B., Checa, A. G., Devaere, L., and Porter, S. M. (2015). To infer the early evolution of mollusc shell microstructures. Key Eng. Mater. **672,** 113-133. DOI: 10.4028/www.scientific.net/KEM.672.113.

67. Li, L., Zhang, X., Yun, H., and Li, G. (2017). Complex hierarchical microstructures of Cambrian mollusk *Pelagiella*: insight into early biomineralization and evolution. Sci. Rep. **7, 1935**. DOI:10.1038/s41598-017-02235-9.

68. Vendrasco, M. J., Li, G., Porter, S. M., and Fernandez, C. Z. (2009). New data on the enigmatic *Ocruranus–Eohalobia* group of Early Cambrian small skeletal fossils. Palaeontology **52 (6),** 1373–1396. DOI: 10.1111/j.1475-4983.2009.00913.x.

69. Li, L., Zhang, X., Skovsted, C. B., Yun, H., Pan, B., and Li, G. (2018). Homologous shell microstructures in Cambrian hyoliths and molluscs. Palaeontology **62 (4),** 515-532. DOI: 10.1111/pala.12406.

70. Vinther, J., and Nielsen, C. (2005). The Early Cambrian *Halkieria* is a mollusc. Zool. Scr. **34 (1),** 81−89. DOI: 10.1111/j.1463-6409.2005.00177.x.

71. Yun, H., Zhang, X., Brock, G. A., Li, L., and Li, G. (2021). Biomineralization of the Cambrian chancelloriids. Geology **49 (6),** 623–628. DOI: 10.1130/G48428.1.

72. Porter, S. M. (2008). Skeletal microstructure indicates chancelloriids and halkieriids are closely related. Palaeontology **51 (4),** 865-879. DOI: 10.1111/j.1475-4983.2008.00792.x.

73. Morris, S. C., and Menge, C. (1991). Cambroclaves and Paracarinachitids, early skeletal problematica from the Lower Cambrian of South China. Palaeontology **34 (2),** 357-397.

74. Morris, S. C., Crampton, J. S., Bing, X., Chapman, A. J. (1997). Lower Cambrian Cambroclaves (*incertae sedis*) from Xinjiang, China, with comments on the morphological variability of sclerites. Palaeontology **40 (1),** 167-189.

75. Harvey, T. H. P., Dong, X., and Donoghue, P. C. J. (2010). Are palaeoscolecids ancestral ecdysozoans? Evol. Dev. **12,** 177-200. DOI: 10.1111/j.1525-142X.2010.00403.x.

76. Ortega-Hernández, J. (2015). Lobopodians. Curr. Biol. **25,** R873-R875.
