# Supplementary material for: Ediacaran Corumbella has a cataphract calcareous skeleton with controlled biomineralization
Source: iScience. 2022 Nov 25;25(12):105676. doi: 10.1016/j.isci.2022.105676 (PMC9763863; doi:10.1016/j.isci.2022.105676)
Supplement: Data S1. Supplemental text [file mmc5.pdf]

The midline and the tube polyhedral section are valid morphological features

Corumbella is built by articulated rings proximally that grade to articulated plates organised in a polyhedral mode. Plates are discontinuous and alternate at either sides of the midline, which is located at the center of each face, following the length of the tube. Midline follows twisted and bended shapes of the tubes (Figs. S1; S3A; Figs. 5B; 6F of Pacheco et al.[1]). Such features support the midline as a valid morphological feature *contra* Walde et al.[2]. Post-burial fractures are common taphonomic artifacts usually misinterpreted as midlines in *Corumbella*[3] (Fig. S1). However, fractures are usually irregular and restricted to specific locations in *Corumbella* (Fig. S1) and other Ediacaran tubular fossils, such as *Conotubus* (see Cai et al.[4]). Fractures do not present the characteristic zigzagged pattern of midlines neither follow tube length; they rather abruptly crosscut the plates (Fig. S1).

There is a variation of morphology along the tube, that is an uniseriate proximal region grades to a quadriseriate distal region with midline; the proximal region has circular cross-section that grades to a polyhedral cross-section distally[1,5]. The validity of a polyhedral cross-section was contested on the grounds that it would be a taphonomic feature[2], but we rather consider it as a valid morphological feature (Fig. 2F; Fig. 3A of Pacheco et al.[1]). For example, fossils with both circular and polyhedral tube parts are preserved in a single sample, and the transition of both morphologies is noted in a single fossil (Figs. S1B; S5A; Fig. 6F of Pacheco et al.[1]), which are both evidence for the presence of a true polyhedral part of the *Corumbella* tube.

Comparison of *Corumbella* skeleton crystallography with other taxa

As for *Corumbella* sclerite crystallography, anthozoan sclerites/spicules possess co-oriented calcite c-axis that is parallel to the sclerite/spicule main axis and to its surface[6] (see Vielzeuf et al.[7] for a more complex pattern). However, as the distribution of sclerites/spicules can be more random, the crystallographic orientation is not consistent among sclerites/spicules, and thus neither relative to the body main axis, as suggested for *Corumbella*. Microfabrics with a c-axis crystallographic orientation (or fibrous crystals) parallel to the biomineralized structure surface are not exclusive from anthozoans. Isopod crustaceans also possess calcite c-axis co-oriented parallelly to the tergite surface but aligned to the bilateral symmetry plan[8]. Craniid brachiopods also have calcite c-axis generally parallel to the shell surface[9]. Aragonite fibres parallel to the sclerite surface and co-oriented to its main axis also occur in halkieriids and Cambroclaves[10], for example. Cambrian molluscs have aragonite fibres parallel to the shell surface[11].

The microstructure of annelid polychaete skeletons

The cataphract skeletal architecture of *Corumbella* is not found in polychaetes. The mode of tube construction of *Corumbella* was compared with modern annelids, like Polychaeta siboglinids[2]. However, these annelids have neither mineralised tubes nor these are formed by two layers of independent building elements like in *Corumbella* but are continuous instead (Table S4). Mineralized tubes of siboglinids have been reported, but mineral growth occurs either by the interaction with microorganisms[12], or post-mortem[13], thus not being controlled by the animal in neither case. When the microstructure of the aragonitic shells of other Polychaeta annelids is considered, further differences are noted (Table S4). In sabellids, the continuous organo-aragonitic wall is double-layered, formed by an outer spherulitic layer and an internal layer of spherulitic prysms, both layers being separated by organic layers[14]. In cirratulitids, aragonitic shells have continuous lamellar walls and internal tabulae[15], and the microstructure is like that of the inner layer of sabeliids[14]. Serpulids also possess single-layered continuous tubes that may be formed by a biologically controlled process[16]. The skeleton has organic-rich and mineral-rich laminae, and is composite, aragonitic or composed by calcite[16]. Serpulid tubes commonly have irregularly oriented prisms, as well as horizons of co-oriented crystals juxtaposed to horizons with co-aligned crystals, but in a different orientation from the

neighbouring horizons, yielding the 'chevron' arrangement of crystal fabrics[14] (see Vinn et al.[16] for a complete description of serpulid mineral textures).

Geological Setting and Age

The Corumbá Group consists of 600 m of mixed siliciclastic-carbonate succession occurring within the South Paraguay Belt, western Brazil, on the eastern border of the Rio Apa Block[17] (Fig. S2). The Tamengo Formation, upper portion of the Corumbá Group, is comprised of shales, mudstones, wackestones, packstones and grainstones that record shallow to relatively deep marine storm-dominated environment[18-20] (Fig. S2). Recent U-Pb ages of 542.27 ± 0.38 Ma and 541.85 ± 0.75 Ma in zircon grains from ash beds and $\delta^{13}C$ isotopic excursion with values ranging from -5 ‰ and +5.5 ‰ indicate a late Ediacaran depositional age for the Tamengo Formation[18,21,22]. Moreover, this unit hosts a diverse terminal Ediacaran fossil record, including vendotaenids, conulariids[23], acritarchs[24], meiofaunal ichnofossils[21], the shelly fossil *Cloudina lucianoi*[25-27], and the focus of this manuscript, *Corumbella werneri*[5,28]. *Corumbella werneri* is found in low-energy settings within silty-shale facies, in the outer to distal mid-ramp[20].

Supplemental references list

1. Pacheco, M. L. A. F., Leme, J. M., and Machado, A. F. (2011). Taphonomic analysis and geometric modelling for the reconstitution of the Ediacaran metazoan *Corumbella werneri* Hahn et al. 1982 (Tamengo Formation, Corumbá Basin, Brazil). J. Taphonomy*.* **9 (4),** 269–283.

2. Walde, D. H.-G., Weber, B., Erdtmann, B.-D., and Steiner, M. (2019). Taphonomy of *Corumbella werneri* from the Ediacaran of Brazil: sinotubulitid tube or conulariid test? Alcheringa **43 (3),** 335–350. DOI: 10.1080/03115518.2019.1615551.

3. Sampaio, G. (2018). Paleobiologia de *Corumbella werneri* (Ediacarano, Grupo Corumbá): Implicações Paleoecológicas e Evolutivas, dissertation, Universidade Federal de São Carlos, São Carlos, SP.

4. Cai, Y., Schiffbauer, J. D., Hua, H., and Xiao, S. (2011). Morphology and paleoecology of the late Ediacaran tubular fossil *Conotubus hemiannulatus* from the Gaojiashan Lagerstätte of southern Shaanxi Province, South China. Prec. Res. **191 (1-2),** 46-57. DOI: 10.1016/j.precamres.2011.09.002.

5. Pacheco, M. L. A. F., Galante, D., Rodrigues, F., Leme, J. M., Bidola, P., Hagadorn, W., Stockmar, M., Herzen, J., Rudnitzki, I. D., Pfeiffer, F., and Marques, A. C. (2015). Insights into the skeletonization, lifestyle, and affinity of the unusual Ediacaran fossil *Corumbella*. PLoS ONE. DOI: 10.1371/journal. pone.0114219.

6. Kingsley, R. J., and Watabe, N. (1982). Ultrastructural investigation of spicule formation in the gorgonian *Leptogorgia virgulata* (Lamarck) (Coelenterata: Gorgonacea). Cell Tissue Res. **223 (2),** 325-334. DOI: 10.1007/BF01258493.

7. Vielzeuf, D., Floquet, N., Perrin, J., Tambutté, E., and Ricolleau, A. (2017). Crystallography of complex forms: the case of octocoral sclerites. Cryst. Growth Des., Am. Chem. Soci. **17 (10),** 5080-5097. DOI: 10.1021/acs.cgd.7b00087.

8. Seidl, B. H. M., Reisecker, C., Hild, S., Griesshaber, E., and Ziegler, A. (2012). Calcite distribution and orientation in the tergite exocuticle of the isopods *Porcellio scaber* and

Armadillidium vulgare (Oniscidea, Crustacea) – a combined FE-SEM, polarized SCµ-RSI and EBSD study. Z. Kristallogr. **227,** 777–792. DOI: 10.1524/zkri.2012.1567.

9. Pérez-Huerta, A., England, J., and Cusack, M. (2007). Crystallography of craniid brachiopods by electron backscatter diffraction (EBSD). Earth Environ. Sci. Trans. R. Soc. Edinb. **98,** 437-442. DOI: 10.1017/S1755691007079832.

10. Porter, S. M. (2008). Skeletal microstructure indicates chancelloriids and halkieriids are closely related. Palaeontology **51 (4),** 865-879. DOI: 10.1111/j.1475-4983.2008.00792.x.

11. Li, L., Zhang, X., Yun, H., and Li, G. (2017). Complex hierarchical microstructures of Cambrian mollusk *Pelagiella*: insight into early biomineralization and evolution. Sci. Rep. **7, 1935**. DOI:10.1038/s41598-017-02235-9.

12. Rincón-Tomás, B., Somoza, L., Sauter, K., Hause-Reitner, D., Madureira, P., Schneider, D., González, F. J., Medialdea, T., Carlsson, J., Reitner, J., and Hoppert, M. (2019). New insights into Siboglinidae microbiota – external tube contributes to an increment of the total microbial biomass. Preprint at https://peerj.com/preprints/27730/.

13. Haas, A., Little, C. T. S., Sahling, H., Bohrmann, G., Himmler, T., and Peckmann, J. (2009). Mineralization of vestimentiferan tubes at methane seeps on the Congo deep-sea fan. Deep-Sea Res. Part I: Oceanogr. Res. Pap. **56 (2),** 283–293. DOI: 10.1016/j.dsr.2008.08.007.

14. Vinn, A., Ten Hove, H. A., and Mutvei, H. (2008). On the tube ultrastructure and origin of calcification in Sabellids (Annelida, Polychaeta). Palaeontology **51 (2),** 295–301. DOI: 10.1111/j.1475-4983.2008.00763.x.

15. Fischer, R., Pernet, B., and Reitner, J. (2000). Organomineralization of Cirratulid Annelid tubes - Fossil and Recent examples. Facies **42,** 35-50. DOI: 10.1007/BF02562565.

16. Vinn, A., Ten Hove, H. A., Mutvei, H., and Kirsimäe, K. (2008). Ultrastructure and mineral composition of serpulid tubes (Polychaeta, Annelida). Zool. J. Linnean Soc. **154 (4),** 633–650. DOI: 10.1111/j.1096-3642.2008.00421.x.

17. Alvarenga, C. J. S., Moura, C. A. V., Gorayeb, P. S. S., and Abreu, F. A. M. (2001). Paraguay and Araguaia belts. In Tectonic Evolution of South America, U. G. Cordani, E. J. Milani, A. T. Filho, and D. A. Campos, eds. (Sociedade Basileira de Geologia), pp. 183-193.

18. Boggiani, P. C., Gaucher, C., Sial, A. N., Babinski, M., Simon, C. M., Riccomini, C., Ferreira, V. P., and Fairchild, T. R. (2010). Chemostratigraphy of the Tamengo Formation (Corumbá Group, Brazil): a contribution to the calibration of the Ediacaran carbon-isotope curve. Prec. Res. **182 (4),** 382-401. DOI: 10.1016/j.precamres.2010.06.003.

19. Oliveira, R. S., Nogueira, A. C. R., Romero, G. R., Truckenbrodt, W., and Bandeira, J. C. S. (2019). Ediacaran ramp depositional model of the Tamengo Formation, Brazil. J. S. Am. Earth Sci. **96,** 102348. DOI: 10.1016/j.jsames.2019.102348.

20. Amorim, K B., Afonso, J. W. L., Leme, J. M., Diniz, C. Q. C., Rivera, L. C. M., Gómez-Gutiérrez, J. C., Boggiani, P. C., and Trindade, R. I. F. (2020). Sedimentary facies, fossil distribution and depositional setting of the late Ediacaran Tamengo Formation (Brazil). Sedimentology **67 (7),** 3422-3450. DOI: 10.1111/sed.12749.

21. Parry, L. A., Boggiani, P. C., Condon, D. J., Garwood, R. J., Leme, J. M., McIlroy, D., Brasier, M. D., Trindade, R., Campanha, G. A. C., Pacheco, M. L. A. F., Diniz, C. Q. C., and Liu, A. G. (2017). Ichnological evidence for meiofaunal bilaterians from the terminal Ediacaran and earliest Cambrian of Brazil. Nat. Ecol. Evol. **1**. DOI: 10.1038/s41559-017-0301-9.

22. Nogueira, A. C. R., Romero, G. R., Sanchez, E. A. M., Domingos, F. H. G., Bandeira, J., Santos, I. M. dos, Pinheiro, R. V. L., Soares, J. L., Lafon, J. M., Afonso, J. W. L. et al. (2019). The Cryogenian-Ediacaran boundary in the Southern Amazon Craton. In Chemostratigraphy Across Major Chronological Boundaries, A. N. Sial, C. Gaucher, M. Ramkumar, and V. P. Ferreira, eds. (American Geophysical Union), pp. 89-114.

23. Van Iten, H., Leme, J. M., Pacheco, M. L. A. F., Simões, M. G., Fairchild, T.R., Rodrigues, F., Galante, D., Boggiani, P. C., and Marques, A. C. (2016). Origin and early diversification of phylum Cnidaria: key macrofossils from the Ediacaran system of North and South America. In The Cnidaria, Past, Present and Future: The World of Medusa and Her sisters, S. Goffredo and Z. Dubinsky, eds. (Springer), pp. 31-40.

24. Gaucher, C., Boggiani, P. C., Sprechmann, P., Sial, A. N., and Fairchild, T. R. (2003). Integrated correlation of the Vendian to Cambrian Arroyo del Soldado and Corumba Groups (Uruguay and Brazil). Palaeogeographic, palaeoclimatic and palaeobiologic implications. Prec. Res. **120 (3-4),** 241-278. DOI: 10.1016/S0301-9268(02)00140-7.

25. Adorno, R. R., Carmo, D. A. do, Germs, G., Walde, D. H. G., Denezine, M., Boggiani, P. C., Sousa e Silva, S. C., Vasconcelos, J. R., Tobias, T. C., Guimarães, E. M. et al. (2017). *Cloudina lucianoi* (Beurlen and Sommer, 1957), Tamengo Formation, Ediacaran, Brazil: taxonomy, analysis of stratigraphic distribution and biostratigraphy. Prec. Res. **301,** 19-35. DOI: 10.1016/j.precamres.2017.08.023.

26. Becker-Kerber, B., Pacheco, M. L. A. F., Rudnitzki, I. D., Galante, D., Rodrigues, F., Leme, J. M. (2017). Ecological interactions in *Cloudina* from the Ediacaran of Brazil: implications for the rise of animal biomineralization. Sci. Rep. **7, 5482**. DOI: 10.1038/s41598-017-05753-8.

27. Becker-Kerber, B., Silva, F. R. da, Amorim, K. B., Pacheco, M. L. A. F., and Leme, J. M. (2020). Putting the cart before the horse: an example of how the lack of taphonomical approaches can mislead paleobiological inferences for the late Ediacaran. Prec. Res. **332**, 105385. DOI: 10.1016/j.precamres.2019.105385.

28. Fairchild, T. R., and Zaine, M. F. (1987). Novas considerações sobre os fósseis da Formação Tamengo, Grupo Corumbá, SW Brasil. Resumo das Comunicações, X Congresso Brasileiro de Paleontologia, Sociedade Brasileira de Paleontologia, Rio de Janeiro, RJ, Brazil, 54-55.
